# Life is viewed as better for minorities in places with more variable habitats

**DOI:** 10.1371/journal.pone.0322084

**Published:** 2025-05-08

**Authors:** Evert Van de Vliert, Mohsen Joshanloo, Lucian G. Conway III, Esther S. Kluwer, Paul A. M. Van Lange

**Affiliations:** 1 Department of Social and Organizational Psychology, University of Groningen, Groningen, The Netherlands; 2 Department of Psychology, Keimyung University, Daegu, South Korea; 3 Department of Psychology, Grove City College, Grove City, United States of America; 4 Department of Social, Health, and Organisational Psychology, Utrecht University, Utrecht, The Netherlands; 5 Behavioral Science Institute, Radboud University, Nijmegen, The Netherlands; 6 Department of Experimental and Applied Psychology, Vrije Universiteit Amsterdam, Amsterdam, The Netherlands; Ain Shams University, EGYPT

## Abstract

Places differ in how livable they are perceived to be for minority groups. But why? We pursue an explanation through the lens of natural habitat variability (varying day length, temperature, and daily precipitation over the course of the year). Uncertainty reduction theory, flexible systems theory, and climato-economic theory offer different explanations for how habitat variability influences mindsets about racial and ethnic minorities, gays and lesbians, foreign immigrants, and people with intellectual disabilities. To test our hypotheses, we analyzed the perceived livability of the place of residence for these minority groups by 1,332,558 native inhabitants from 163 countries. Our results support the theoretical notion that variable habitats foster flexible psychosocial systems. Minorities are viewed to have better livability in places with more variable habitats. Economic affluence reinforces this trend, and the interaction effect is mediated by the quality of governance. These country-level findings (*R*^*2*^ ≈ 0.52) demonstrate construct, concurrent, convergent, divergent, substantive, and forecast validity. They significantly overshadow effects of individual-level characteristics and mindsets (*R*^*2*^ ≈ 0.03). Habitat equations predicting perceived local livability for minorities during one period (2010–2015) forecast up to 75 percent of the extent to which minorities in each of the four hemispheres of the Earth are perceived to be living in a good place at a subsequent period (2016–2020).

## Introduction

**Fig 1:**
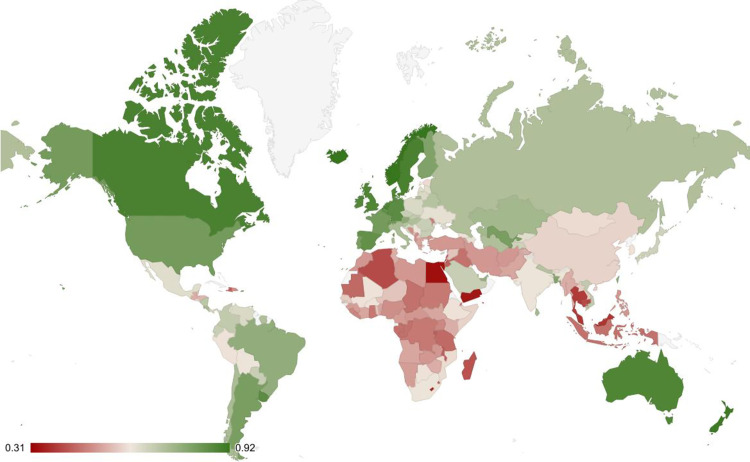
World map of perceived livability for minorities. Perceived livability ranges from very bad (dark red) to very good (dark green). This map covers 163 countries and is based on perceived livability for racial and ethnic minorities, gays and lesbians, foreign immigrants, and people with intellectual disabilities. It was generated using Google sheets.

Perceptions of livability vary from place to place. Here, perceived livability is defined as the habitual assessment of residents’ quality of life within a distant physical habitat and the immediate societal environment that tends to follow. We focus on perceived livability for minority groups because these groups are especially vulnerable to place-bound threats and setbacks to their daily quality of life. For the same reason, the Gallup World Polls [1] assess how people customarily judge the suitability of their own place of residence as a place to live for racial and ethnic minorities, gays and lesbians, foreign immigrants, and intellectually disabled people (see Fig 1 for an overall world map). Popular media often explain these perceived livability differences in superficial terms, identifying prejudice and discrimination as causes. To find a more fundamental explanation, we aim to understand *why* residents adopt specific mindsets about minority groups. What factors might lead people to believe that their own place of residence is or is not a good place to live for such vulnerable minorities?

We begin addressing this question by “zooming out” to examine the broader, pervasive factors influencing experiences of place-bound livability. One of the most fundamental characteristics of any residential location is its global positioning relative to the sun, which dictates variations in sunlight, temperature, and daily precipitation over the course of the year. Thus, the current study investigates the potential connection between perceived local livability for minorities and local variabilities in day length (short, long), temperature (cold, heat), and daily precipitation (drought, deluge). In the following section, we explore the geographical, ecological, and methodological foundations of this study. To better capture and convey the complexity of the issue, we first present a visual model for describing, explaining, and forecasting perceived livability for minorities.

## Foundations

### Overall research model

**Fig 2:**
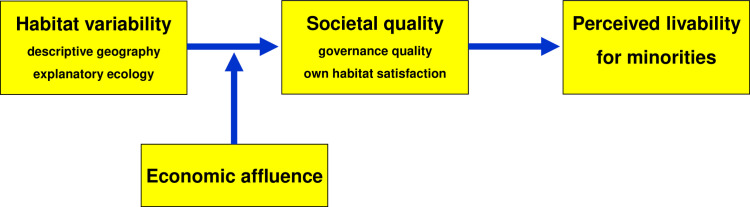
The complex natural ecology of perceived livability.

Our premise is that all living species on Earth must adapt most fundamentally to variabilities in day length, temperature, and daily precipitation over the course of the year. These variabilities increase as one moves from the equator towards the poles and are relatively stable from east to west [2–5]. Humans are no exception. They are highly sensitive to deviations from constant day length, average temperature, and fixed wet and dry seasons. Humans need protection from extreme temperature fluctuations, rely on the seasonal availability of plants and animals for food, and must navigate health risks posed by an ever-changing natural environment. Based on these biogeographical considerations, we propose that the greater habitat variability at higher latitudes influences perceived local livability, particularly affecting the perceived livability for vulnerable minority groups. Fig 2 outlines the proposed relationships between habitat variability, economic affluence, societal quality, and perceived livability for minorities, providing an overview of the content in the following subsections.

### Descriptive geography

The leftmost box of Fig 2 represents the axiom that habitat variability has both a descriptive geographical aspect and an explanatory ecological aspect. Geographically, individuals living at higher latitudes (e.g., Canadians, Norwegians, and New Zealanders) experience more variable—and therefore more stressful—natural conditions compared to those at lower latitudes (e.g., Haitians, Congolese, and Indonesians). It results in a U-shaped distribution of stressful habitat variability from the North to the South Pole, to which the inhabitants of the Earth must adapt. If this stressful variability influences habitual perceptions and judgments, we should observe a corresponding U-shaped (or inverted U-shaped) distribution of these responses between the poles. For the targeted problem, this reasoning led us to expect a U-shaped (or inverted U-shaped) distribution of perceived livability for minority groups between the poles, contrasting with negligible east-west differences in perceived livability for minorities.

This descriptive geographical foundation of our study, though uncommon, is valuable for validation and theorization. Well-documented adaptations [5–10] to natural environments—such as ecological threats, subsistence possibilities, or pathogen prevalence—are rarely linked to latitude and longitude. While geographical positioning serves as a preliminary explanatory tool, it can help validate and refine the hypothesized place-boundness of perceived livability perceptions. One additional refinement is to establish the uniqueness of the geographical distribution of perceived livability for minorities by comparing it with, and relating it to, the geographical distribution of perceived livability for oneself (see *own habitat satisfaction* in the central box of Fig 2). Is there also a U-shaped (or inverted U-shaped) distribution of people’s satisfaction with their place of residence shared with minority groups?

### Explanatory ecology

Racial and ethnic groups, gays and lesbians, foreign immigrants, and intellectually disabled people often face prejudice and discrimination [11,12]. However, local prejudice and discrimination provide only a narrow and superficial explanation of perceived local livability for minorities. The complex natural ecology of perceived livability, illustrated in Fig 2, offers a broader and more fundamental explanation of residents’ livability assessments. Habitat variability is believed to enhance societal quality, which manifests in a sustainable, equitable, and satisfactory living environment. Governance quality, as a political pillar, and own habitat satisfaction, as a psychosocial pillar, are both expected to be associated with the perceived livability for minority groups. We will now discuss three theories that explain the hypothesized path from natural habitat variability through societal quality to perceived local livability for minorities. Two of these theories offer competing predictions, potentially reconciled by the third theory, which proposes economic affluence as a modifier of the impact of habitat variability on societal quality and residents’ livability assessments.

Uncertainty reduction theory [13,14] posits that people are fundamentally motivated to reduce stressful uncertainty in their perceptions and behaviors. To manage uncertainty, individuals may become more rigid (non-adaptable, neurotic), and/or favor ingroup members over outgroup members [13,14]. In the current context, this theory suggests that reducing uncertainty inherent to variability in day length, temperature, and daily precipitation may lead to lower societal quality, manifesting as increased ingroup favoritism and outgroup derogation. Consequently, individuals may perceive better local livability for themselves than for others. This ingroup-outgroup bias in livability perceptions is likely stronger for negatively stereotyped minority groups due to subconscious ingroup identification and outgroup discrimination. It leads to a clear hypothesis: higher uncertainty inherent in greater habitat variability will be associated with less favorable views of local livability for minority groups, mediated by lower societal quality. Controlling for other natural uncertainties (e.g., natural disasters) and alternative mediators from pathogenic diseases and demographic diversity (e.g., ethnic diversity, religious diversity, immigration rate) would strengthen the empirical evidence.

Systems theory [15–17] states that to survive and thrive, systems must adjust their internal flexibilities to the stressful variabilities of their external environments. Stable environments require fixed and routine adjustments (e.g., agrarian communities in fertile valleys), whereas dynamic environments necessitate flexible and creative adjustments (e.g., nomadic tribes in turbulent surroundings) [18–20]. Applied to the present case, systems theory hypothesizes that the numerous and continuous dynamics of more variable habitats are more effectively managed when inhabitants adopt flexible mindsets. Minorities may benefit the most because flexibility as a societal quality implies that majority members adopt less rigidly negative stereotypes, and minority members have easier access to resources (e.g., information, skills), practices (e.g., tasks, evaluations), and payoffs (e.g., wages, prestige). In short, increases in flexibility imply increases in livability for minority groups. Contrary to uncertainty reduction theory, flexible systems theory would be supported if greater habitat variability were associated with more—rather than less—favorable views of local livability for minority groups, mediated by higher societal quality.

The climato-economic theory of freedom (including freedom from prejudice and discrimination) [21,22] predicts that economic affluence modifies the effects of stressful habitat variability. Stressful ecological demands are considered a double-edged sword. Greater demands with insufficient resources increase closed-mindedness and social isolation, whereas greater demands with sufficient resources increase open-mindedness and social connection [22]. Applied to our case, climato-economic theory presents a similar double-edged sword with opposite effects on societal quality and perceived livability for minorities. In societies with limited monetary resources, stressful habitat variability is inherently threatening and is expected to decrease governance quality, own habitat satisfaction, and perceived local livability for minorities. Conversely, in wealthier societies, the same stressful habitat variability provides challenges that can be successfully met, thus increasing governance quality, own habitat satisfaction, and perceived local livability for minorities. Importantly, a proper test of this interaction effect requires controlling for main effects of covariates of societal wealth, such as non-agricultural subsistence, urbanization, population density, and income inequality.

The comparative tests reported below evaluated the theoretical model visualized in Fig 2. Are minority groups perceived to cope with worse societal quality and local livability in places with more variable habitats (uncertainty reduction theory), or to enjoy better societal quality and local livability in such places (flexible systems theory)? These empirical tests also examined whether there is an element of truth in both theories. Specifically, minorities in poorer societies are perceived to be worse off in more variable and thus more threatening habitats, whereas minorities in richer societies are perceived to be better off in more variable and thus more challenging habitats (climato-economic theory of freedom).

### Methodological foundations

The descriptive geography and explanatory ecology of the perceived livability for minorities were tested with nationally representative survey data from 934,682 randomly selected native inhabitants of 163 countries, surveyed between 2010 and 2015. The resulting regression models were further validated by predicting future perceptions of local livability for minorities, based on data from 397,876 different native inhabitants of 152 countries, surveyed between 2016 and 2020. Country was the primary unit of analysis, not only because nations are prominent organizing units for cross-cultural research, but also because habitat variability and other within-country conditions affect all inhabitants similarly. After briefly describing the outcome variable, this subsection discusses the stepwise approach used to test the proposed descriptive and explanatory links between shared locality and perceived livability.

The Gallup World Polls [1] asked representatively and randomly recruited native inhabitants to evaluate the local livability for typically negatively stereotyped minorities. Specifically, respondents indicated whether their city or area of residence is a good place or not a good place to live for racial and ethnic minorities, gay or lesbian people, immigrants from other countries, and people with intellectual disabilities. We analyzed the four binary responses as a composite possessing construct validity in terms of actual group prejudice and discrimination (see Methods section). This approach also reflects the reality that global natural habitats tend to be associated with a holistic mindset, which is more than the sum of its specific parts [23]. The respondent’s own satisfaction with living in the same city or area was not included in this holistic measure of livability perceptions. Instead, it was used as a mediating variable, representing the subjective level of livability that may serve as a baseline criterion for judging the livability for minorities.

We conducted separate tests of the descriptive geography and explanatory ecology of perceived livability for minorities for four reasons. First, the psychometric description of the link between shared locality and perceived livability must precede substantive explanation. Second, absolute latitude and habitat variability are nearly identical variables [*r*_(161)_ = 0.927], causing multicollinearity issues when interpreting descriptive versus explanatory results. Third, controlling for confounding variables makes geographical link descriptions less accurate but improves ecological link explanations. Combining the descriptive and explanatory analyses would therefore create a potentially unclear picture. Finally, separate tests allow for comparisons of descriptive and explanatory forecasts for future perceptions of local livability for minorities. In consequence, we performed descriptive modelling, explanatory modelling, and prospective modelling in a stepwise fashion.

In the first step, descriptive modelling using latitude and longitude established the concurrent, convergent and divergent validity of the hypothesized psychometric link between habitual residence and the perceived Gestalt of local livability for minorities. The repeatability of the latitudinal gradient of perceived livability for minorities across hemispheres indicates concurrent validity. The reversibility of the north-south gradients of perceived livability for minorities in the opposite northern and southern hemispheres indicates convergent validity [5]. The negligibility of east-west gradients of perceived livability for minorities indicates divergent or discriminant validity. Although these psychometric validities are fundamental, they remain descriptively geographical rather than substantively ecological, as habitat variability is implicit rather than explicitly measured and evaluated.

In the second step, we evaluated the three explanatory hypotheses regarding the complex natural ecology of perceived livability for minorities. This step involved two stages. First, we used habitat variability, economic affluence, and societal quality to predict the society-level perception of livability for minorities, as shown in Fig 2. These analyses aimed to assess the substantive validity of habitat variability’s direct effects, as well as its modified and mediated impacts on perceived livability for minorities. Next, we applied multi-level modelling to demonstrate that individual perceptions of livability for minorities are more closely associated with the natural variability of the place of residence than with personal characteristics and mindsets. Finally, prospective modelling generalized the results of both the descriptive and explanatory regressions by incorporating data from new respondents in later years [24–26]. This society-level analysis validated the extent to which the descriptive geographical and explanatory ecological models based on data from 2010–2015 could forecast perceived local livability for minorities between 2016 and 2020.

## Results

### Descriptive geography

In this pre-explanatory analysis, we validated the hypothesized place-boundness of perceived livability for minorities in terms of latitude rather than longitude (see Methods section). The native country of residence was represented by the midrange degree of latitude (negative values south of the geographical equator at 0° N, and positive values north of it) and the midrange degree of longitude (negative values west of the Greenwich meridian at 0° E, and positive values east of it). To capture the U-shaped distribution of perceived livability for minorities between the North and South Poles, and to compute the near-equatorial reversal point of the expected north-south gradient, latitude was standardized and also squared. Longitude was standardized but not squared, as no linear or curvilinear relationship was expected between the east-west axis of the Earth and livability perceptions [5,23].

Data collected between 2010 and 2015 were used to assess the perceived livability for minorities. The sample included 934,682 native inhabitants from 163 countries (ranging from a minimum of 392 to a maximum of 30,936 respondents per country; *M *= 5,734; s.d. = 3,625). Country-level livability perceptions were regressed against latitude and longitude (see S1 Table in S1 File for raw scores on latitude, longitude, and livability). Latitude [*B*_(159)_ = 0.07; *P *< 0.001; *R*^*2*^ = 0.09; confidence interval (CI) = 0.05 to 0.09], squared latitude [*B*_(159)_ = 0.06; *P *< 0.001; *R*^*2*^ = 0.22; CI = 0.04 to 0.07], and longitude [*B*_(159)_ = -0.02; *P *= 0.016; *R*^*2*^ = 0.02; CI = -0.04 to -0.00] together accounted for 33% of the variation in perceived livability for minorities (or 32% after excluding the nine largest countries with the least accurate latitude and longitude measures). The standardized beta coefficients indicate that for every one standard deviation increase in latitude [*B*_(159)_ = 0.49] and squared latitude [*B*_(159)_ = 0.50], perceived livability for minorities increases by 0.49 and 0.50 standard deviations, respectively.

**Fig 3:**
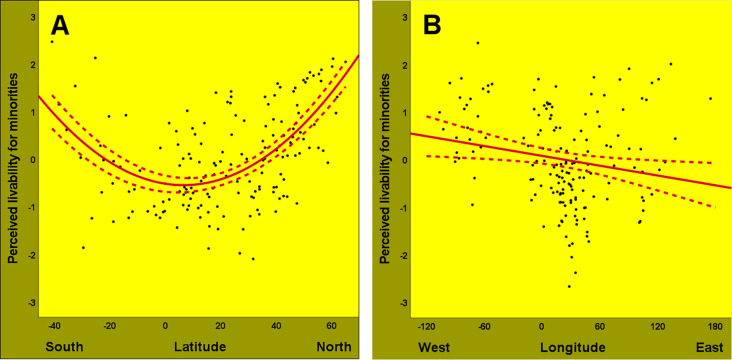
Geographical distributions of perceived livability for minorities. Panel A shows that perceived livability for minorities (*z*) increases at higher latitudes with greater habitat variability, while controlling for longitude. These poleward increases have opposite north-south directions in the ecologically opposite northern (*B*_(129)_ = 0.10; *P *< 0.001; *R*^*2*^ = 0.23; CI = 0.07 to 0.13) and southern (*B*_(28)_ = -0.18; *P *< 0.001; *R*^*2*^ = 0.33; CI = -0.27 to -0.08) hemispheres. In contrast, panel B indicates that perceived livability for minorities (*z*) shows minimal variation along longitude [*B*_(159)_ = -0.02; *P *= 0.016; *R*^*2*^ = 0.03; CI = -0.04 to -0.00], while controlling for latitude. The broken lines represent confidence intervals (95%).

Fig 3 shows that life is perceived as better for minorities at higher latitudes in both the northern (*R*^*2*^ = 0.23) and southern (*R*^*2*^ = 0.33) hemispheres. This pattern was replicated when analyzed separately in the eastern (*R*^*2*^ = 0.33) and western (*R*^*2*^ = 0.52) hemispheres, with higher effect sizes due to the extra contribution of squared latitude (see S2 Table in S1 File for details). In addition, as detailed in S3 Table in S1 File, the U-shaped curve depicted in Fig 3 is consistent across racial and ethnic minorities (*R*^*2*^ = 0.07), gays and lesbians (*R*^*2*^ = 0.38), foreign immigrants (*R*^*2*^ = 0.15), and intellectually disabled people (*R*^*2*^ = 0.36). Indeed, the perceived livability for various minorities increases towards the poles across all hemispheres (concurrent validity), shifts in opposite north-south directions in the ecologically opposite northern and southern hemispheres (convergent validity), and remains relatively stable across the east-west axis (divergent validity). It is difficult to attribute this specific geographical distribution of perceived livability for minorities to spatial differences in scaling problems (given the binary response format) or to spatial autocorrelation (see Methods section).

To explore the uniqueness and significance of these results, we repeated the analysis for perceived livability for oneself, expressed as own habitat satisfaction—the percentage of respondents satisfied with their city or area while living alongside minority groups (see S1 Table in S1 File for values). Although the geographical distribution of own habitat satisfaction mirrored that of perceived livability for minorities, the results were less pronounced. Latitude [*B*_(159)_ = 4.20; *P *< 0.001; *R*^*2*^ = 0.07; CI = 2.23 to 6.17], squared latitude [*B*_(159)_ = 2.07; *P *< 0.02; *R*^*2*^ = 0.04; CI = 0.47 to 3.66], and longitude [*B*_(159)_ = -1.44; *P *= 0.12; *R*^*2*^ = 0.01; CI = -0.39 to 3.28] accounted for only 12% of the variation in own habitat satisfaction. Comparing the results reveals that the U-shaped latitudinal gradient is considerably steeper for perceived local livability for minorities (*R*^*2*^ = 0.31) than for respondents’ own habitat satisfaction (*R*^*2*^ = 0.11). This perceptual difference implies that the livability of the same residential location is judged and viewed differently for minority groups than for oneself, likely because one’s own habitat satisfaction is relatively free from prejudice. Direct confirmation of this speculation is not possible because the Gallup World Polls do not explicitly assess prejudice, as it is considered too sensitive an issue.

The speculative hypothesis that the U-shaped latitudinal gradient in perceived livability for minorities may be influenced by prejudice is supported by a similar U-shaped gradient in discrimination between “we-groups” and “they-groups” [11,23]. Notably, this gradient for intergroup discrimination (*R²* = 0.54) is even steeper than that for perceived livability for minorities (*R²* = 0.31). If our hypothesis holds, it may be informative to observe that the latitudinal gradients for racial and ethnic minorities (*R²* = 0.07) and foreign immigrants (*R²* = 0.15) more closely resemble the less prejudice-prone gradient for respondents’ own habitat satisfaction (*R²* = 0.12). In contrast, the steeper gradients for gays and lesbians (*R²* = 0.38) and for the intellectually disabled (*R²* = 0.36) align more closely with the gradient for intergroup discrimination (*R²* = 0.54). Could this suggest an increasing degree of prejudice across groups, from racial and ethnic minorities, to foreign immigrants, to the intellectually disabled, and then to gays and lesbians?

### Explanatory ecology

The explanatory analyses assumed that inhabitants of the same country, exposed to similar habitat variability, tend to share perceptions of local livability for minority groups. To test the more detailed natural ecology of perceived livability for minorities (see Fig 2), estimates of day-length variability, temperature variability, and daily-precipitation variability were factor analyzed into a composite score of a country’s habitat variability. National wealth was measured as the average log-transformed income per capita in international dollars at the beginning of this century [27–29]. Indicators for habitat variability, national wealth, and governance quality are reported in S4 Table in S1 File. Own habitat satisfaction and perceived livability for minorities were measured the same as in the preceding analysis.

**Fig 4:**
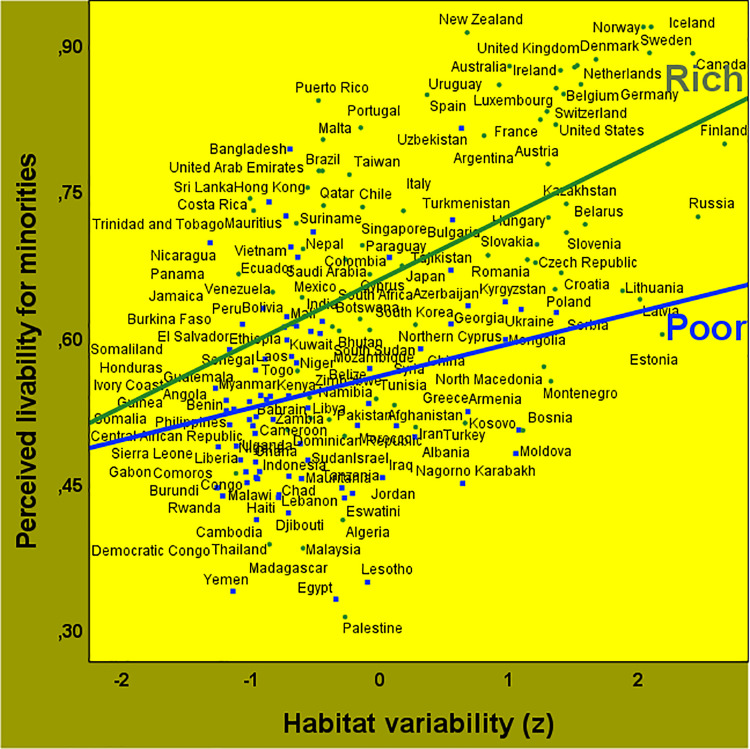
Effects of habitat variability on perceived livability for minorities in poor and rich societies. Perceived local livability increases in more variable habitats: *r*_(161)_ = 0.55; *P *< 0.001; CI = 0.43 to 0.65. The increases are larger for richer societies [*r*_(77)_ = 0.47; *P *< 0.001; CI = 0.28 to 0.63] than for poorer societies [*r*_(82)_ = 0.23; *P *= 0.04; CI = 0.01 to 0.42; *z *= 1.76; *P *= 0.04; total *R*^*2*^ = 0.50].

We started testing the interaction effect because economic affluence might suppress the main effect of habitat variability on perceived livability for minorities. Together, habitat variability [*B*_(159)_ = 0.02; *P *= 0.037; *R*^*2*^ = 0.30; CI = 0.00 to 0.04], national wealth [*B*_(159)_ = 0.08; *P *< 0.001; *R*^*2*^ = 0.17; CI = 0.06 to 0.10], and their interaction [*B*_(159)_ = 0.03; *P *< 0.01; *R*^*2*^ = 0.03; CI = 0.01 to 0.05] accounted for 50% of the societal variation in perceived livability for minorities (or 47% after excluding the nine largest countries). Habitat variability had a substantially larger effect than national wealth. As visualized in Fig 4, the perceived local livability for minorities increased in more variable habitats, especially in richer societies (for a breakdown by larger and smaller hemispheres, see S5 Table in S1 File; for a breakdown by day-length variability, temperature variability, and daily-precipitation variability, see S6 Table in S1 File). These main and interaction effects of habitat variability on perceived livability for minorities generalized across minority groups (for statistical and visual details, see S7 Table and S1–S4 Figs in S1 File).

Mediation analysis revealed that the impact of habitat variability on perceived livability for minorities [zero-order *r*_(161)_ = 0.55; *B*_(158)_ = 0.025; *P *< 0.01; CI = 0.007 to 0.043] is partially mediated by governance quality [*B*_(158)_ = 0.026; bootstrap CI = 0.012 to 0.045] and own habitat satisfaction [*B*_(158)_ = 0.013; CI = 0.006 to 0.021], rather than national wealth [*B*_(158)_ = 0.012; bootstrap CI = -0.004 to 0.026]. Thus, national wealth acts as a modifier rather than a mediator. Subsequent conditional process analysis, detailed in Table 1, showed that habitat variability has a direct effect on perceived livability for minorities [*B*_(159)_ = 0.029; *P *< 0.01; CI = 0.011 to 0.047], as well as a wealth-modified effect that is mediated by governance quality [index of modified mediation = 0.012; CI = 0.007 to 0.017] rather than own habitat satisfaction [index of modified mediation = 0.002; CI = -0.003 to 0.008] (total *R*^*2*^ = 0.57). These wealth-modified mediation effects also generalized across minority groups, with total *R*^*2*^ values ranging from 0.18 to 0.66 (*P*_*s*_< 0.001; see S8–S11 Tables in S1 File). As discussed later, the effect sizes varied due to the differing direct effects of habitat variability and own habitat satisfaction.

**Table 1:** Explanatory ecological model: main and interaction effects of habitat variability on perceived livability for minorities mediated by governance quality rather than own habitat satisfaction (*R*^*2*^ = 0.57; *N *= 163)^a^.

Predictor	*B*	s.e.	*P*	LLCI	ULCI
Habitat variability (HV)	0.029	0.009	< 0.001	0.011	0.047
Governance quality	0.058	0.009	0.018	0.039	0.076
Own habitat satisfaction	0.003	0.001	0.008	0.002	0.005
Conditional indirect effect of habitat variability via governance quality (index of modified mediation = 0.012, CI = 0.007 to 0.017)					
Predictor	*B*	Boot s.e.		LLCI	ULCI
Poor (NW -1 s.d.)	-0.010	0.005		-0.022	0.000
Intermediate (mean NW)	0.004	0.004		-0.004	0.011
Rich (NW + 1 s.d.)	0.017	0.004		0.010	0.026
Conditional indirect effect of habitat variability via own habitat satisfaction (index of modified mediation = 0.002, CI = -0.003 to 0.008)					
Predictor	*B*	Boot s.e.		LLCI	ULCI
Poor (NW -1 s.d.)	-0.002	0.006		-0.015	0.009
Intermediate (mean NW)	0.000	0.004		-0.008	0.008
Rich (NW + 1 s.d.)	0.003	0.003		-0.003	0.010

^a^A highly similar pattern of results was obtained for each of the targeted minority groups (see S8–S11 Tables in S1 File).

These results support the substantive validity of the complex natural ecology of perceived livability for minorities, in part because the relatively prejudice-free measure of respondents’ own habitat satisfaction was taken into consideration as a comparison baseline. Contrary to the uncertainty reduction theory and in line with the flexible systems theory, livability is generally perceived as better for minorities in more variable habitats. Additionally, there is partial support for the climato-economic theory of freedom, as increases in perceived livability for minorities are larger in relatively rich societies compared to poorer societies (Fig 4; S1–S4 Figs in S1 File). However, contrary to climato-economic theorizing, perceived livability for minorities was generally higher among the relatively poor living in more variable habitats. A systems-theoretical explanation of this unexpected finding is that more variable cold habitats have historically fostered greater flexibility through economic development [5,30–33], leading to more favorable livability views among the relatively poor in more variable habitats.

The suggested alternative mediators and other covariates were added to the basic interaction model in two steps. First, ecological covariates of habitat variability (natural disasters, pathogenic diseases, agricultural subsistence, urbanization) showed no effects beyond the interaction effects of habitat variability and national wealth on perceived livability for minorities (S12 Table in S1 File). Second, potential demographic covariates of livability for minorities (population density, ethnic diversity, religious diversity, immigration rate, income inequality) also did not affect the findings (S13 Table in S1 File). The lack of significant impact from these variables implies that governance quality remains a major, uncontested mediator of the empirical connection between habitat variability and the perceived local livability for minorities, while accounting for the generally experienced satisfaction of living in the targeted city or area.

### Multi-level modelling

The analyses above assume that perceptions of local livability for minorities are a societal rather than individual phenomenon, primarily shaped by the natural variability of the residential area. This assumption was tested using multi-level analysis, comparing the predictive power of the society-level factors shown in Fig 2 with the predictive power of personal characteristics and mindsets derived from the Gallup World Polls [1]. Individual predictors of individual-level perceptions of livability for minorities included gender, age, secondary education, college education, household income adequacy, and psychosocial flourishing. The individual respondent’s relatively prejudice-free habitat satisfaction was a particularly relevant predictor, as it represents the baseline criterion against which the perceived local livability for minority groups must be adjusted. Specifically, we regressed the individual-level perceptions of livability for minorities on (1) seven individual-level predictors, (2) two country-level mediators: governance quality and own habitat satisfaction, and (3) three ultimate country-level predictors: habitat variability, national wealth, and their interaction.

The results, summarized in Table 2, indicate that perceptions of livability for minorities are associated with individual factors in the following order (by increasing standardized estimates): education, age, gender, income adequacy, psychosocial flourishing, and one’s own habitat satisfaction (*R*^*2*^ ≈ 0.03). Country-level factors had a larger influence, with habitat variability, national wealth, their interaction, and aggregated own habitat satisfaction as significant predictors (*R*^*2*^ ≈ 0.52). Post-hoc mediation analyses further revealed that the main and interaction effects of habitat variability on perceptions of livability for minorities are not mediated by governance quality or respondents’ own habitat satisfaction. In contrast, own habitat satisfaction mediates the effect of national wealth (see the next-to-last row of Table 2). Overall, these findings suggest that country-level effects substantially outweigh individual characteristics and mindsets in shaping perceived local livability for minorities.

**Table 2:** Individual-level effects (*R*^*2*^ ≈ 0.03) and country-level effects (*R*^*2*^ ≈ 0.52) on individual perceptions of livability for minorities.

Level^a^	Predictor^b^	Unstandardized estimate	Posterior s.d.	95% confidence interval (CI)		*P*	Standardized estimate
				Lower	Upper		
1	Female	0.015	0.001	0.014	0.016	< 0.001	0.023
1	Age	0.044	0.002	0.040	0.049	< 0.001	0.022
1	Secondary education	0.001	0.001	0.000	0.003	0.080	0.002
1	College education	0.007	0.001	0.004	0.009	< 0.001	0.008
1	Income adequacy	0.014	0.000	0.014	0.015	< 0.001	0.039
1	Psychosocial flourishing	0.113	0.002	0.109	0.116	< 0.001	0.070
1	Own habitat satisfaction	0.102	0.001	0.100	0.103	< 0.001	0.133
2	Habitat variability (HV)	0.021	0.010	0.002	0.041	0.014	0.164
2	National wealth (NW)	0.041	0.013	0.015	0.066	< 0.001	0.353
2	HV * NW	0.016	0.008	0.001	0.031	0.022	0.137
2	Governance quality (GQ)	0.004	0.014	-0.022	0.032	0.375	0.035
2	Own habitat satisfaction (OHS)	0.274	0.074	0.129	0.419	< 0.001	0.254
M	HV → GQ → PLM	0.000	0.001	-0.002	0.004	0.402	
M	NW → GQ → PLM	0.003	0.009	-0.015	0.021	0.375	
M	(HV * NW) → GQ → PLM	0.001	0.002	-0.004	0.006	0.375	
M^c^	HV → OHS → PLM	0.002	0.003	-0.004	0.009	0.237	
M^c^	NW → OHS → PLM	0.015	0.005	0.006	0.025	< 0.001	
M^c^	(HV * NW) → OHS → PLM	0.000	0.002	-0.004	0.005	0.400	

^a^Level 1 is the individual level (*N *= 934,682), level 2 is the country level (*N *= 163), and M refers to the country-level mediation of country-level effects on individual perceptions of livability for minorities (PLM). ^b^ Predictors refer to the main effects, 1 interaction effect (HV * NW), and 6 indirect effects (last six rows). The effects are posterior median point estimates. ^c^ OHS is not significantly predicted by HV and HV * NW; however, NW significantly predicts OHS (0.054; *P *< 0.001; CI = 0.036 to 0.072; standardized effect = 0.506; *R*^*2*^ = 0.314).

### Prospective modelling

Can the descriptive geographical and explanatory ecological models also predict the future degree of perceived livability for minorities? We answered this question using more recent data from different samples of inhabitants. From 2016 onward, the Gallup World Polls [1] have continued to ask whether the residential city or area is a good place or not a good place to live for racial and ethnic minorities, gay or lesbian people, and immigrants from other countries (excluding people with intellectual disabilities). We used the resulting proportional scores of perceived livability for minorities from 2016 to 2020 (0–3 times *a good place to live*) as a criterion to test the predictive power of models developed on the preceding 2010–2015 period. We investigated the robustness of forecast accuracy for each of the four hemispheres, enhancing the comparability of hemispheric forecasts by adjusting the latitudinal position of the equator [34] (see Methods section).

For the descriptive geographical model, the 2010–2015 equation [*Y* = (0.070 * *z*-latitude) + ((0.059) * (*z*-latitude * *z*-latitude)) + (-0.023 * *z*-longitude)] could forecast about 18% of the worldwide distribution of perceived livability for minorities along latitude rather than longitude. This forecast power is robust across hemispheres (see Table 3; S14 Table in S1 File for details). Similarly, the explanatory ecological model, using the 2010–2015 equation [*Y* = (0.023 * *z*-habitat variability) + (0.078 * *z*-national wealth) + ((0.028) * (*z*-habitat variability * *z*-national wealth))] could forecast 28% to 75% of the country-level variation in perceived local livability for minorities within the four hemispheres (see Table 3; S14 Table in S1 File for details). Table 3 clearly shows that the explanatory ecological model has higher forecast power than the descriptive geographical model, both worldwide and in each of the four hemispheres.

**Table 3:** Forecast accuracy of perceived livability for minorities in the future (2016-2020) based on two regression models developed in the past (2010-2015).

Global area	Hemisphere				Entire world
	North	South	East	West	
Sample size					
Number of countries	117	35	114	38	152
Number of respondents	313,087	84,789	300,298	97,578	397,876
Predictive power^a^					
Descriptive geography^b^	0.412 (0.249 to 0.552)	0.508 (0.211 to 0.720)	0.310 (0.134 to 0.468)	0.786 (0.622 to 0.883)	0.430 (0.291 to 0.551)
Explanatory ecology^c^	0.582 (0.447 to 0.690)	0.713 (0.498 to 0.845)	0.530 (0.383 to 0.650)	0.864 (0.753 to 0.928)	0.594 (0.481 to 0.688)

^a^Shown are correlations with 95% confidence intervals (*P*_*s*_< 0.001) between the forecasted and measured degrees of perceived livability for minorities. ^b^Descriptive forecast based on the 2010–2015 equation (*R*^*2*^ ≈ 0.33): *Y* = (0.070 * *z*-latitude) + ((0.059) * (*z*-latitude * *z*-latitude)) + (-0.023 * *z*-longitude). ^c^Explanatory forecast based on the 2010–2015 equation (*R*^*2*^ ≈ 0.50): *Y* = (0.023 * *z*-habitat variability) + (0.078 * *z*-national wealth) + ((0.028) * (*z*-habitat variability * *z*-national wealth)).

## Discussion

Our central empirical finding is that minorities worldwide are perceived to have better livability in places at higher latitudes with greater habitat variability over the course of the year. This latitudinal gradient of perceived livability for minorities is valid because it (1) represents actual group prejudice and discrimination (construct validity); (2) is replicated across hemispheres (concurrent validity); (3) has opposite north-south directions in the ecologically opposite northern and southern hemispheres (convergent validity); (4) contrasts with negligible east-west differences in habitat variability and in perceived livability for minorities (divergent validity); (5) can be explained by stressful variabilities in day length, temperature, and daily precipitation (substantive validity); and (6) can be predicted in advance (forecast validity). The distinct main effect of habitat variability is partially influenced by economic affluence, and this interaction effect is robust when controlling for various societal conditions and generalizes across different minority groups. Perceived livability for racial and ethnic minority members, gays and lesbians, foreign immigrants, and the intellectually disabled increases the most among wealthier people living in more variable habitats at higher latitudes.

These data largely support the systems theoretical explanation [15–20] that greater flexibility is needed in more variable habitats. Our results align with the idea that locals develop more flexible mindsets, including more open-mindedness towards local minority groups, under conditions of more stressful variabilities in day length, temperature, and daily precipitation. The results also support the theoretical idea from climato-economic theory [21,22], that the impact of more variable habitats on flexibility towards minority groups is reinforced by higher economic wealth, as collective cash and capital amplify flexible courses of action for everyone. The observed wealth-modified impact of habitat variability on governance quality, own habitat satisfaction, and perceived livability for minorities complements theories about simple main effects of economic [5,30–33] and institutional [33] development on collective psychosocial functioning. Furthermore, the variability-flexibility explanation may offer new perspectives on established theories, such as the tightness-looseness theory of culture [35,36].

Tight societies are characterized by strict social norms and harsh punishments for deviation, whereas loose societies are characterized by more flexible social norms and greater tolerance for deviant behavior [35]. Our findings suggest that life is perceived as better for minorities in looser cultures situated in more variable habitats at higher latitudes. The geographical distributions of preindustrial and contemporary hierarchism and collectivism support this speculation: tight, vertically collectivist cultures peak at the equator and taper off towards the North and South Poles [11]. Conversely, loose, horizontally individualist cultures increase in more variable habitats at higher latitudes. Similarly, our present findings indicate that tight autocratic versus loose democratic governance mediates the wealth-modified impact of greater habitat variability on the perceived better livability for minorities at higher latitudes.

The variability-flexibility explanation also informs Berry’s eco-cultural framework [37]. His model examines how background variables, like ecological and socio-political contexts, influence process variables, including cultural practices and societal structures, which ultimately shape psychological variables. While the complex natural ecology of mindsets and practices [5,23], as depicted for perceived livability in Fig 2, shares a similar conceptual structure, it introduces two fundamental geographical and ecological innovations. From a geographical perspective, the natural ecology model underscores that inhabitants’ mental and behavioral habits in physical habitats can be meaningfully related to latitude, longitude, and equatorial reversals of north-south habit gradients [5]. Ecologically, this model emphasizes that inhabitants’ mental and behavioral habits are linked to habitat variability—notably differences in day length, temperature, and daily precipitation—and consequently to variabilities in plant and animal life. It also incorporates a fresh viewpoint on the biotic ecology of prejudice and discrimination [11].

Consider, for example, the rice-wheat theory, which argues that due to the intensity and reciprocity of labor exchanges, the differentiation and discrimination between ingroups and outgroups became stronger in rice regions than in wheat regions [7,8]. The present findings might suggest a complementary explanation: rice habitats are less variable than wheat habitats, leading to more outgroup discrimination and worse livability for minority groups. Similarly, the pathogen-stress theory posits that inhabitants of regions with higher pathogen prevalence became more collectivistic and ethnocentric to avoid potentially disease-carrying strangers [9,10]. The present findings might suggest that higher pathogen prevalence and greater habitat stability both contribute to increased outgroup discrimination and worse livability for minority groups. In both theories, annual habitat variability provides a complementary explanation to annual average temperature, with the rationale being that warm-blooded humans respond primarily to deviations from the annual average temperature [22].

Indeed, our explanatory ecological model reveals that pathogenic diseases do not uniquely affect perceived livability for minorities beyond the wealth-dependent effects of habitat variability. Instead, though overshadowed by the more immediate mediating effect of governance quality, pathogen prevalence may serve as an additional mediator of the interaction effect shown in Fig 4. Regardless of interpretation, the results imply that purely economic, political, or psychosocial explanations of perceived livability for minorities are incomplete. Such explanations might falsely suggest that the economic, political, and psychosocial layers of the environment are not embedded within layers of varying biotic and abiotic habitats. Importantly, purely economic, political, and psychosocial explanations—including personal, interpersonal and intergroup explanations [12,14]—fail to account for the opposite north-south gradients of perceived livability for minorities above and below the thermal equator at 6° N [34], and for the relatively negligible east-west gradients of perceived livability for minorities (Fig 3).

The wealth-dependent explanation of perceived livability for minorities in terms of habitat variability is no exception to the general rule that a theory is stronger when it meets the criteria of parsimony, generality, and accuracy [38–40]. However, no theory can perfectly satisfy all three. Any explanation that fulfills two of these criteria often struggles to meet the third. In this study, the parsimony and generality are achieved by focusing on single variables. Habitat variability is the common denominator of deviations from constant day length, average temperature, and stable wet and dry seasons, which in turn affect continuous crop growth and pathogen stress throughout the year [2–5,22]. Perceived livability for minorities is the common denominator of all the positively viewed elements of the natural, institutional and interpersonal environment.

Further, the climato-economic theory of freedom, while achieving parsimony and accuracy, sacrifices generality by making the effects of more variable habitats partially dependent on national wealth. Finally, the criterion of generality is most clearly met by applying uncertainty reduction [14] and flexible systems [15–20] theories to the variability of abiotic habitats and the adjustments required by their inhabitants. The wealth-dependent path leads from greater habitat variability through increased flexibility in free and effective governmental functioning, to more favorable perceptions of local livability for members of relatively unfamiliar minority groups.

Focusing on society-level pathways from global natural habitats to the Gestalt of perceived livability for minorities has yielded significant insights. However, the wealth-dependent paths for the four minority groups vary in effect size due to additional direct effects (see S8–S11 Tables in S1 File). Specifically, the direct positive effects of habitat variability and own habitat satisfaction are considerably larger for psychosocially closer outgroups (intellectually disabled people, gays and lesbians) than for psychosocially more distant outgroups (racial and ethnic minorities, foreign immigrants). A likely, yet speculative, explanation is that the local observers’ judgments of livability are more prejudiced against psychosocially closer outgroups, such as intellectually disabled people and gays and lesbians (see descriptive results). Other groups that might fall into this category could include the elderly, the physically handicapped, drug addicts, and people with a criminal record. Further research is needed to clarify the relative importance of direct and indirect effects of habitat variability in shaping the relationship between majority and minority groups, both across countries and within large nations such as Australia, Brazil, China, Russia, and the United States.

Despite the strength of Gallup’s annual representative sampling, our inferences are limited to 934,682 world citizens surveyed between 2010 and 2015. Nevertheless, we compared the ability of our descriptive and explanatory regression models to forecast future perceptions of livability for minorities (Table 3). These forecasts reveal that country-level perceptions of livability for minorities are best predicted by the interaction between habitat variability and national wealth, as outlined in the climato-economic theory of freedom [21,22]. Indeed, the wealth-reinforced association between habitat variability and country-level perceptions of minority livability before 2016 provided the strongest predictor of perceived minority livability for the 2016–2020 period, both worldwide [*r*_(150)_ = 0.59; *P *< 0.001] and across the northern, southern, eastern, and western hemispheres [0.53 < *r*_(150)_ < 0.86; *P*_*s*_< 0.001]. The differences in forecast power across hemispheres should be interpreted with caution, as the Greenwich meridian is not an equator and the hemispheres vary significantly in their range of inhabited latitudes as well as in the number and size of countries.

The results from the cross-level and cross-time analyses suggest that individual observers play a modest role in the overall construction and persistence of perceived local livability for minorities. Individuals deprived of adequate household income, psychosocial flourishing, or own habitat satisfaction have only a minor negative effect on their perception of local livability for minorities. However, the reverse may also be worth exploring: more negative mindsets about minority groups might be found not only among those experiencing relative deprivation but also among those who feel relatively gratified [41]. Previous research has suggested that fear of societal decline among wealthier individuals is linked to opposition to immigration, and by extension, lower perceived livability for immigrants. This raises an important question for future studies: Does insecure prosperity also reduce favorable perceptions of livability for other minorities in more variable habitats?

In summary, the explainability and predictability of the association between greater habitat variability and more favorable perceptions of livability for minorities underscore the need for broad transdisciplinary theories that explore the complex pathways from natural habitats to natural mental and behavioral habits. Many proximal explanations for mindsets and practices can be enriched by considering their broader context, including variabilities in day length, temperature, and daily precipitation. Efforts to reduce social inequality could benefit from focusing on people deprived of economic, political, or psychosocial resources, particularly inhabitants of more stable habitats (see Figs 1 and 2). Thus, this study emphasizes that alongside the proximal influences of institutions and interpersonal dynamics, the more distal influences of natural habitats are essential to understanding the development and persistence of cultures that either promote equality for all groups—or fail to do so.

Almost all of the above has overarching implications for the existential urgency of climate change. Due to global warming, the greater decreases in temperature variability and daily-precipitation variability at higher latitudes [42,43] are likely to pull inhabitants of currently more variable habitats away from flexible and creative mental and behavioral habits, instead pushing them towards fixed and routine habits. Consequently, global warming may increase gradual changes towards more rigid societies with increased intergroup discrimination, thereby undermining fundamental freedoms globally. Thus, climate change can bring about diverse challenges, including not only increases in temperature and natural hazards, but also mindsets that pose risks to the social livability of minority groups around the world. With more evidence in support of this claim, we recommend to help mitigate global warming by including such psychosocial consequences in educational programs and climate action projects.

## Methods

### General information

We used country as the primary unit of analysis because inhabitants of a country largely share the same habitat variability and socio-economic, governmental, and judicial systems. We retained the nine countries with the largest north-south variations in habitat variability (Argentina, Australia, Brazil, Canada, Chile, China, India, Russia, and the United States) as removing them had only a negligible effect on the results. In total, data were available for 163 countries surveyed between 2010 and 2015 (N = 934,682; tests of the descriptive and explanatory models), and for 152 countries surveyed between 2016 and 2020 (N = 397,876; tests of the forecast power of the descriptive and explanatory models).

Gallup gathers data through cross-sectional annual surveys of about one thousand representatively and randomly recruited native inhabitants from a varying number of countries each year. Our sample size was increased by combining within-country responses across years. In countries where telephone coverage represents at least 80% of the population, Gallup uses telephone surveys, employing a random-digit-dial method or a nationally representative list of phone numbers. In other countries, face-to-face interviews are conducted by local interviewers, who randomly select one adult household member for the interview.

Below, we present overviews of the measures of perceived livability for minorities, the main predictors, and the society- and individual-level variables. We then outline the analytical steps, progressing from descriptive geographical modelling to explanatory ecological and multi-level modelling, and finally to prospective modelling. SPSS 25.0 was used for inferential statistics in the country-level tests of the descriptive geographical and explanatory ecological models. Bayesian modeling was conducted using Mplus version 8.7 for the tests of the multi-level model. The analysis scripts used are provided in the Supporting information, S1–S3 Methods in S1 File. Confidence intervals (CIs) reported are 95% CIs.

### Perceived livability for minorities

Respondents were asked four questions with a binary response format: “Is the city or area where you live a good place or not a good place to live for (1) racial and ethnic minorities; (2) gay or lesbian people; (3) immigrants from other countries; and (4) people with intellectual disabilities?” Each individual’s response—0–4 times *a good place*—served as a proportional (0–1) score for livability perceptions (0.52 < *r*_*t*_ < 0.64; KR21 = 0.65; *M *= 0.62; s.d. = 0.35). At the country level, 22 missing values (3.37%) were imputed using the missForest algorithm [44] from the R package missRanger [45], which estimates the missing part from the non-missing part of the data. Country-level perceptions of livability for minorities (see S1 Table in S1 File) range from 0.31 for Palestinians and 0.33 for Egyptians to 0.92 for Icelanders and Norwegians (M = 0.62; s.d. = 0.14), follow a normal distribution (skewness = 0.29; s.e. = 0.19; kurtosis = -0.68; s.e. = 0.38), and are internally consistent (0.52 < *r* < 0.80; Cronbach’s α = 0.84).

Eliminating response biases when asking respondents worldwide to evaluate elements of the natural, institutional, and interpersonal environments of negatively stereotyped groups is challenging. The Gallup Organization addresses this challenge in five ways. First, data are collected using standardized procedures across different languages, with questions translated and back-translated to ensure linguistic equivalence. Second, a binary response format is used to reduce cultural differences in extremity rating. Third, foreign-born respondents are excluded as they may have less accurate perceptions of local livability for minority groups. Fourth, no direct questions are asked about sensitive attitudes towards negatively stereotyped groups. Finally, respondents serve as impartial local informants rather than simply representing themselves.

We tested the construct validity of our outcome measure of perceived local livability for minorities by correlating it at the country level with measures related to group prejudice and discrimination. Consistent with our expectations, the country-level measure of perceived local livability for minority groups is negatively related to positive discrimination of ingroups [21] [*r*_(153)_ = -0.62; *P *< 0.001; CI = -0.71 to -0.52], negative discrimination through legal restrictions on the rights to sexual freedom, abortion, and the right of criminals to remain alive [46] [*r*_(158)_ = -0.59; *P *< 0.001; CI = -0.69 to -0.49], and social exclusion of ethnic, religious, and economic minority groups [44] [*r*_(125)_ = -0.67; *P *< 0.001; CI = -0.76 to -0.57].

### Main predictors

#### Latitude and longitude.

The geographical coordinates of each country’s midrange latitude and midrange longitude were retrieved from https://developers.google.com/public-data/docs/canonical/countries_csv and reproduced in S1 Table in S1 File. The latitude of countries (N = 163; M = 21.29; s.d. = 24.45), approximated a normal distribution (skewness = -0.45; s.e. = 0.19; kurtosis = -0.46; s.e. = 0.38) and provided an adequate representation of world territories larger than 300 km^2^ [Δ*M* = -2.82, *t*_(211)_ = -1.67, *P *= 0.10, CI = -6.15 to 0.50].

#### Habitat variability.

The raw data and summary statistics of the country-level variables for day-length variability, temperature variability, and daily-precipitation variability over the course of the year are reported in S4 Table in S1 File. Habitat variability is the common factor of the standardized variabilities in day length, temperature, and daily precipitation (eigenvalue λ = 2.19, *R*^*2*^ = 0.73; Cronbach’s α = 0.81; M = 0.12; s.d. = 1.03; skewness = 0.74; s.e. = 0.19; kurtosis = -0.57; s.e. = 0.38). Day-length variability is the absolute difference between day-length hours on June 21 and December 21, computed by the Chronology Unit of the University of Groningen based on the country’s midrange latitude [2]. Temperatures and precipitation are measured across each country’s major cities, weighted for population size [47]. Temperature variability is the standard deviation of four average temperatures—the lowest and highest temperatures in the coldest and hottest months [47]. Daily-precipitation variability, which increases with the extent that both dry and wet months are exceptions, is approximated by the minimal monthly precipitation divided by the maximal monthly precipitation [47].

We validated the resulting estimate of habitat variability across modern countries by comparing it to a measure of climate variability across 583 pre-industrial societies. Botero et al. [48] identified a gradient of increasing societal exposure to more variable temperatures throughout the year and less predictable annual cycles of temperature and precipitation from 1901 to 1950. Absolute latitude [*B*_(580)_ = 0.046; *P *< 0.001; CI = 0.043 to 0.050] and longitude-linear [*B*_(580)_ = 0.001; *P *= 0.109; CI = -0.000 to 0.001] accounted for 58% of the climate variability across the 583 societies. We used this regression equation to predict our more comprehensive measure of habitat variability, which included day-length variability across 163 modern countries. The strong positive correlation between predicted and measured habitat variability [*r*_(160)_ = 0.922; *P *< 0.001; CI = 0.895 to 0.942] confirms the validity of our estimate of general habitat variability.

#### National wealth.

National wealth, measured with the recommended time lag of over a decade to allow causality to take hold, consisted of the average of the logged income per head in 2000, 2002, and 2004 [27–29] (M = 8.53; s.d. = 1.17; skewness = -0.02; s.e. = 0.19; kurtosis = -1.03; s.e. = 0.38; for country scores, see S4 Table in S1 File).

### Society-level variables

Details regarding the sources and descriptive statistics of the country-level mediating and control variables can be found in S15 Table in S1 File, and details regarding their correlations with the outcome variable and predictors are in Table 4. The aggregate measure of the respondents’ habitat satisfaction, modelled as a mediator, is detailed later under “Individual-level variables.”

**Table 4:** Explanatory ecological model: intercorrelations of the outcome variable (1) and main predictors (2-3) with the mediating (4-5) and control variables (6-15).

	*1*	*2*	*3*
1. Perceived livability for minorities			
2. Habitat variability	0.551*** (0.434 to 0.650)		
3. National wealth (ln)	0.655*** (0.558 to 0.735)	0.599*** (0.491 to 0.690)	
4. Governance quality	0.684*** (0.593 to 0.758)	0.582*** (0.470 to 0.675)	0.794*** (0.729 to 0.844)
5. Own habitat satisfaction	0.571*** (0.458 to 0.667)	0.324*** (0.179 to 0.455)	0.501*** (0.376 to 0.608)
6. Natural disasters (ln)	-0.423*** (-0.546 to -0.281)	-0.503*** (-0.614 to -0.373)	-0.557*** (-0.659 to -0.436)
7. Pathogenic diseases	-0.483*** (-0.592 to -0.355)	-0.723*** (-0.789 to -0.640)	-0.675*** (-0.750 to -0.581)
8. Agricultural subsistence	-0.560*** (-0.657 to -0.444)	-0.586*** (-0.679 to -0.475)	-0.858*** (-0.894 to -0.811)
9. Urbanization	0.480*** (0.353 to 0.590)	0.392*** (0.254 to 0.515)	0.766*** (0.706 to 0.831)
10. Population density	0.106 (-0.049 to 0.255)	-0.056 (-0.208 to 0.098)	0.211** (0.059 to 0.353)
11. Ethnic diversity	-0.345*** (-0.474 to -0.202)	-0.406*** (-0.527 to -0.269)	-0.538*** (-0.639 to -0.419)
12. Religious diversity	0.013 (-0.141 to 0.166)	0.046 (-0.109 to 0.198)	-0.065 (-0.216 to 0.090)
13. Immigration rate	0.140 (-0.014 to 0.288)	0.084 (0.070 to 0.235)	0.273*** (0.125 to 0.410)
14. Income inequality	-0.281*** (-0.427 to -0.121)	-0.573*** (-0.675 to -0.450)	-0.274*** (-0.421 to -0.114)
15. Perceived business opportunities	-0.045 (-0.198 to 0.109)	-0.477*** (-0.588 to -0.349)	-0.274*** (-0.411 to -0.126)

* *P *< 0.05. ** *P *< 0.01. *** *P *< 0.001.

#### Governance quality.

Quality of public governance served as a mediating variable (see Fig 2). Rather than being an objective truth, it is judged as better to the extent that it meets many observers’ subjective standards. Therefore, the World Bank has designed six indicators of perceived governance quality: voice and accountability, political stability, government effectiveness, regulatory quality, rule of law, and control of corruption. Interobserver reliability was improved by integrating observational data from over 30 survey institutes, think tanks, non-governmental organizations, international organizations, and private sector firms. The “voice and accountability” component is particularly relevant here, as it emphasizes respect for the rights and freedoms of minorities, including ethnic, religious, linguistic, and immigrant groups. Nevertheless, we used the full index of all six components because they are so closely interrelated (eigenvalue λ = 5.21, *R*^*2*^ = 0.87; Cronbach’s *α* = 0.97) that they yield almost identical mediator information.

#### Natural disasters.

We measured the relevance of natural disasters in inducing uncertainty by using the logged annual average of affected individuals per million inhabitants. This includes events such as earthquakes, volcanic eruptions, climate extremes, epidemics, insect infestations, and wildfires. Climate extremes and wildfires are especially relevant due to their link with global warming.

#### Pathogenic diseases.

The prevalence of human-to-human transmitted diseases, such as cholera, dengue fever, hookworm, leishmaniasis, leprosy, and measles, is important for two reasons: its relevance as a potential mediator based on the pathogen-stress theory [9,10], and its association with global warming.

#### Agricultural subsistence.

The average percentage of employment in the agrarian sector from 1990–2002 serves as a control variable because this percentage tends to increase in more stable habitats [11], inhabited by poorer populations [30].

#### Urbanization.

The percentage of a country’s population living in urban areas is confounded with both increased habitat variability and greater exposure to outgroups (own data inspection).

#### Population density.

The logged number of inhabitants per square kilometer is considered a potentially confounding determinant of perceived livability of the place of residence.

#### Ethnic and religious diversity.

The probability that two randomly selected inhabitants of a country belong to different ethnic or religious groups may heighten societal uncertainty. These diversity factors are crucial for properly testing the uncertainty reduction theory [13.14].

#### Immigration rate.

The immigration rate, considered as an alternative mediator or confounder, was measured as the annual net migration rate per 1,000 inhabitants for the period 2010–2015. To reduce extreme kurtosis caused by large refugee flows, we capped both inflows (e.g., Lebanon and Qatar) and outflows (e.g., Syria and Central African Republic) at 15% of the population.

#### Income inequality.

Income equality, an important factor for testing the climato-economic theory of freedom [21,22], was measured by assessing the deviation in income distribution among individuals or households from a perfectly equal distribution.

### Individual-level variables

To explore the possible relevance of individual-level effects compared to country-level effects on perceived local livability for minorities, we investigated the influence of five personal characteristics, household income adequacy, and the respondent’s psychosocial flourishing and habitat satisfaction.

#### Personal characteristics.

The Gallup World Polls measure the respondent’s gender (male, female), secondary education (no, yes), and college education (no, yes) as dichotomous variables. Age is measured in years.

#### Income adequacy.

The Gallup World Polls also ask respondents: “Which phrase comes closest to your own feelings about your household’s income these days? (1) Living comfortably on present income; (2) Getting by on present income; (3) Finding it difficult on present income; (4) Finding it very difficult on present income” (reverse coded).

#### Psychosocial flourishing.

Individuals who live a free and meaningful life in a stimulating and supportive social environment tend to be more tolerant towards outgroups [11,12,14]. For that reason, we included the Gallup World Polls’ index of psychosocial flourishing or eudaimonic well-being as a predictor of perceived livability towards minorities. As elaborated in S4 Methods in S1 File, this index includes seven items with a binary response format (eigenvalue λ = 1.63, *R*^*2*^ = 0.23): Are you satisfied or dissatisfied with your freedom to choose what you do with your life? Did you learn or do something interesting yesterday? Can people in this country get ahead by working hard, or not? [In the past month] have you helped a stranger or someone you didn’t know who needed help? [In the past month] have you volunteered your time to an organization? If you were in trouble, do you have relatives or friends you can count on to help you whenever you need them, or not? Were you treated with respect all day yesterday?

#### Own habitat satisfaction.

Participants in the Gallup World Polls [1] are also asked: “Are you satisfied or dissatisfied with the city or area where you live?” The individual-level dichotomy is used as a control variable in the multi-level model, while the country-level average is used as mediating variable in the explanatory ecological model.

### Analytical steps

#### Descriptive geography.

We regressed perceived local livability for minorities in the 163 countries on the linear and squared terms of standardized latitude, and on standardized longitude. The variables were standardized for comparability and comprehensibility: *Y* = (0.070 * *z*-latitude) + ((0.059) * (*z*-latitude * *z*-latitude)) + (-0.023 * *z*-longitude). There were no outliers (Cook’s distances < 0.53). The near-equatorial reversal point of this north-south gradient of livability perceptions was computed as *X*_*m*_ + (-*b*_1_/ 2*b*_2_) = 7.39 degrees or 7°24^’^ N, where *X*_*m *_= the mean midrange latitude of the 163 countries, -*b*_1 _= the regression coefficient for linear latitude (*X*), and *b*_2 _= the regression coefficient for squared latitude (*X*^*2*^) [5]. To check the robustness, generalizability, and uniqueness of the results, we repeated the regression analysis for the northern, southern, eastern, and western hemispheres, for each of the targeted minority groups, and for own habitat satisfaction. To ensure that the north-south gradients of livability perceptions in Fig 3 are not artifacts of spatial autocorrelation, we confirmed that the Durbin-Watson statistics for the northern (1.81) and southern (1.83) hemispheres are close to the midpoint (2) of the 0–4 point criterion scale.

#### Explanatory ecology.

We first regressed perceived local livability for minorities in the 163 countries on standardized habitat variability, standardized national wealth, and their interaction: *Y* = (0.023 * *z*-habitat variability) + (0.078 * *z*-national wealth) + ((0.028) * (*z*-habitat variability * *z*-national wealth)). There was no evidence of problematic outliers (Cook’s distances < 0.09) or spatial autocorrelation (Durbin-Watson statistic = 1.96). Next, we used mediational and conditional process analysis to test the theoretical model visualized in Fig 2. We applied Hayes’s [49] templates 4 and 7, reporting results with heteroscedasticity-corrected standard errors and drawing 5,000 bootstrap samples to construct bias-corrected 95% confidence intervals. The observed main and interaction effects of habitat variability (Fig 4) appeared to be mediated by governance quality rather than own habitat satisfaction. Finally, we computed the basic interaction model while controlling for the effects of alternative mediators and confounds identified in the foundations section.

#### Multi-level modelling.

The individually perceived local livability for minorities was regressed on seven individual-level predictors (gender, age, secondary education, college education, income adequacy, psychosocial flourishing, and own habitat satisfaction) and five country-level predictors [habitat variability (HV), national wealth (NW), HV*NW, governance quality (GQ), and aggregated own habitat satisfaction (OHS)]. Psychosocial flourishing was included as a known confounder of household income and national wealth [50]. Own habitat satisfaction was incorporated as it reflects the respondent’s subjective level of livability, which may serve as a baseline criterion for judging the livability for minorities. All individual-level variables, along with the aggregated measure of own habitat satisfaction, were derived from the Gallup World Polls [1].

A Bayesian estimation approach was used with 5000 draws, with the first half designated as burn-in. The GIBBS (PX1) algorithm was employed across two Markov chain Monte Carlo chains, using Mplus default priors [51]. Posterior distributions were recorded every 30th iteration to thin the chains. Individual-level age, income adequacy, and psychosocial flourishing were group-mean centered [52], while country-level variables were grand-mean centered. The perceived local livability for minorities was regressed on the mediators (GQ and OHS) and the ultimate predictors (HV, NW, HV*NW) at the country level. Potential scale reduction factors [53] indicated the convergence of the model parameters and the quality of the posterior distributions. Bayesian posterior parameter trace plots and autocorrelation plots [51] did not reveal any problems with chain convergence and mixing either.

#### Prospective modelling.

From 2016 to 2020, 397,876 representatively and randomly recruited native inhabitants from 152 countries provided new data on the perceived livability for racial and ethnic minorities, gay and lesbian people, and immigrants from other countries (0.48 < *r*_*t*_ < 0.61; KR21 = 0.54; *M *= 0.65; s.d. = 0.36). This three-item index was used as the criterion variable in regression models aimed at prospectively predicting spatial differences in perceived livability for minorities. The descriptive geographical and explanatory ecological models, based on data from the 2010–2015 period, served as predictors (Table 3). We evaluated the forecast power of these models separately within each of the four hemispheres, adjusting for the latitudinal position of the equator as described below.

It is long known that Earth’s thermal equator lies at 6° N latitude [34], north of the geographical equator (0° N), and that seasonal variations in human mortality increase as distance from the thermal equator grows [34]. Consistent with this knowledge, we observed that the north-south gradients of perceived livability for minorities, as shown in Fig 3, reverse their trajectories—declining versus rising—at the thermal equator (6° N) rather than at the geographical equator (0° N) (see “Descriptive geography” in the Methods section for details). Accordingly, we refined the physical, biological, and psychosocial comparability of the four hemispheres by basing the surface areas of the northern and southern hemispheres, and the hemispheric forecasts, on the thermal equator at 6° N.

## Supporting information

S1 File Four figures (S1-–S4 Figs), fifteen tables (S1–S15 Tables), and four Method files (S1-S4 Methods). **S1 Fig.** Effects of habitat variability on perceived livability for racial and ethnic minorities in poor (blue) and rich (green) societies. Perceived local livability increases in more variable habitats: *r*_(161)_ = 0.283; *P *< 0.001; CI = 0.135 to 0.418. The increases are larger for richer societies [*r*_(77)_ = 0.317; *P *= 0.004; CI = 0.103 to 0.503] than for poorer societies [*r*_(82)_ = 0.031; *P *= 0.782; CI = -0.185 to 0.243; *z *= 1.862; *P *= 0.031; total *R*^*2*^ = 0.137]. **S2 Fig.** Effects of habitat variability on perceived livability for gays and lesbians in poor (blue) and rich (green) societies. Perceived local livability in more variable habitats: *r*_(161)_ = 0.588; *P *< 0.001; CI = 0.478 to 0.681. The increases are larger for richer societies [*r*_(77)_ = 0.490; *P *< 0.001; CI = 0.301 to 0.641] than for poorer societies [*r*_(82)_ = 0.261; *P *= 0.017; CI = 0.049 to 0.450; *z *= 1.684; *P *= 0.046; total *R*^*2*^ = 0.632]. **S3 Fig.** Effects of habitat variability on perceived livability for foreign immigrants in poor (blue) and rich (green) societies. Perceived local livability increases in more variable habitats: *r*_(161)_ = 0.306; *P *< 0.001; CI = 0.160 to 0.439. The increases are larger for richer societies [*r*_(77)_ = 0.353; *P *< 0.001; CI = 0.143 to 0.533] than for poorer societies [*r*_(82)_ = 0.010; *P *= 0.928; CI = -0.205 to 0.224; *z *= 2.247; *P *= 0.012; total *R*^*2*^ = 0.181]. **S4 Fig.** Effects of habitat variability on perceived livability for intellectually disabled people in poor (blue) and rich (green) societies. Perceived local livability increases in more variable habitats: *r*_(161)_ = 0.576; *P *< 0.001; CI = 0.464 to 0.671. The increases are larger for richer societies [*r*_(77)_ = 0.507; *P *< 0.001; CI = 0.322 to 0.655] than for poorer societies [*r*_(82)_ = 0.305; *P *= 0.005; CI = 0.097 to 0.488; *z *= 1.526; *P *= 0.064; total *R*^*2*^ = 0.484]. **S1 Table.** Supplementary data for the descriptive geographical model. **S2 Table.** Descriptive effects of latitude and longitude on perceived livability for minorities (total *N *= 163), broken down by hemisphere. **S3 Table.** Descriptive effects of latitude and longitude on perceived livability for minorities (*N* = 163), broken down by minority group. **S4 Table.** Supplementary data for the explanatory ecological model. **S5 Table.** Explanatory ecological model: main and interaction effects of habitat variability on perceived livability for minorities (total *N *= 163), broken down by hemisphere. **S6 Table.** Explanatory ecological model: main and interaction effects of habitat variability on perceived livability for minorities (*N *= 163), broken down by component of variability. **S7 Table.** Explanatory ecological model: main and interaction effects of habitat variability on perceived livability for minorities (*N *= 163), broken down by minority group. **S8 Table.** Explanatory ecological model: main and interaction effects of habitat variability on perceived livability for racial and ethnic minorities mediated by governance quality rather than own habitat satisfaction (*R*^*2*^ = 0.243; *N *= 163). **S9 Table.** Explanatory ecological model: main and interaction effects of habitat variability on perceived livability for gays and lesbians mediated by governance quality rather than own habitat satisfaction (*R*^*2*^ = 0.664; *N *= 163). **S10 Table.** Explanatory ecological model: main and interaction effects of habitat variability on perceived livability for foreign immigrants mediated by governance quality rather than own habitat satisfaction (*R*^*2*^ = 0.177; *N *= 163). **S11 Table.** Explanatory ecological model: main and interaction effects of habitat variability on perceived livability for people with intellectual disabilities mediated by governance quality rather than own habitat satisfaction (*R*^*2*^ = 0.545; *N *= 163). **S12 Table.** Explanatory ecological model: main and interaction effects of habitat variability on perceived livability for minorities controlled for ecological covariates (*R*^*2*^ = 0.533; *N *= 150)^a^. **S13 Table.** Explanatory ecological model: main and interaction effects of habitat variability on perceived livability for minorities controlled for demographic covariates (*R*^*2*^ = 0.531; *N *= 140)^a^. **S14 Table.** Prospective model: predictors, predictions, and the criterion measure of perceived livability for minorities. **S15 Table.** Explanatory ecological model: descriptive statistics of mediating and control variables, with sources in footnotes.
(DOCX)
