# Supplementary material for: Life is viewed as better for minorities in places with more variable habitats
Source: PLoS One. 2025 May 8;20(5):e0322084. doi: 10.1371/journal.pone.0322084 (PMC12061393; doi:10.1371/journal.pone.0322084)
Supplement: S1 File — Four figures (S1-–S4 Figs), fifteen tables (S1–S15 Tables), and four Method files (S1-S4 Methods). S1 Fig. Effects of habitat variability on perceived livability for racial and ethnic minorities in poor (blue) and rich (green) societies. Perceived local livability increases in more variable habitats: r(161) = 0.283; P < 0.001; CI = 0.135 to 0.418. The increases are larger for richer societies [r(77) = 0.317; P = 0.004; CI = 0.103 to 0.503] than for poorer societies [r(82) = 0.031; P = 0.782; CI = -0.185 to 0.243; z = 1.862; P = 0.031; total R2 = 0.137]. S2 Fig. Effects of habitat variability on perceived livability for gays and lesbians in poor (blue) and rich (green) societies. Perceived local livability in more variable habitats: r(161) = 0.588; P < 0.001; CI = 0.478 to 0.681. The increases are larger for richer societies [r(77) = 0.490; P < 0.001; CI = 0.301 to 0.641] than for poorer societies [r(82) = 0.261; P = 0.017; CI = 0.049 to 0.450; z = 1.684; P = 0.046; total R2 = 0.632]. S3 Fig. Effects of habitat variability on perceived livability for foreign immigrants in poor (blue) and rich (green) societies. Perceived local livability increases in more variable habitats: r(161) = 0.306; P < 0.001; CI = 0.160 to 0.439. The increases are larger for richer societies [r(77) = 0.353; P < 0.001; CI = 0.143 to 0.533] than for poorer societies [r(82) = 0.010; P = 0.928; CI = -0.205 to 0.224; z = 2.247; P = 0.012; total R2 = 0.181]. S4 Fig. Effects of habitat variability on perceived livability for intellectually disabled people in poor (blue) and rich (green) societies. Perceived local livability increases in more variable habitats: r(161) = 0.576; P < 0.001; CI = 0.464 to 0.671. The increases are larger for richer societies [r(77) = 0.507; P < 0.001; CI = 0.322 to 0.655] than for poorer societies [r(82) = 0.305; P = 0.005; CI = 0.097 to 0.488; z = 1.526; P = 0.064; total R2 = 0.484]. S1 Table. Supplementary data for the descriptive geographical model. S2 Table. Descripti [file pone.0322084.s001.docx]

**Supporting Information**

**Life is perceived as better for minorities in places with more variable habitats**

Evert Van de Vliert

Mohsen Joshanloo

Lucian G. Conway III

Esther S. Kluwer

Paul A. M. Van Lange


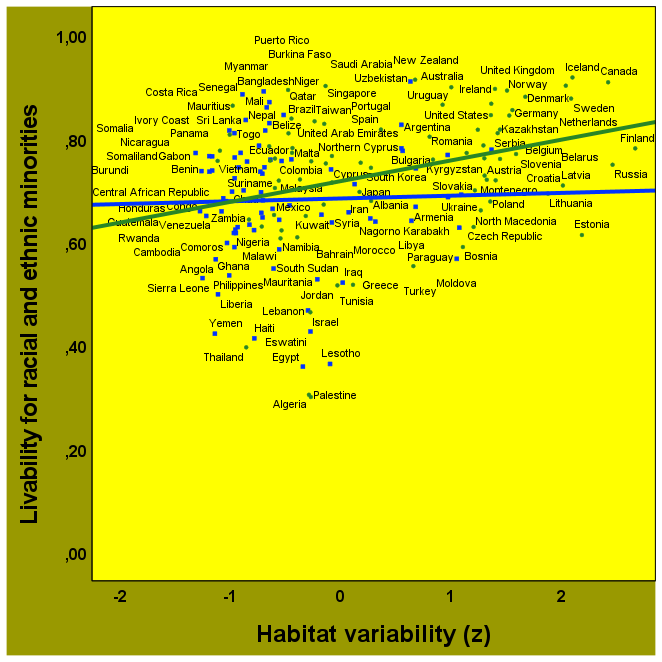


**S1 Fig | Effects of habitat variability on perceived livability for racial and ethnic minorities in poor (blue) and rich (green) societies.** Perceived local livability increases in more variable habitats: *r*_(161)_ = 0.283; *P* < 0.001; CI = 0.135 to 0.418. The increases are larger for richer societies [*r*_(77)_ = 0.317; *P* = 0.004; CI = 0.103 to 0.503] than for poorer societies [*r*_(82)_ = 0.031; *P* = 0.782; CI = -0.185 to 0.243; *z* = 1.862; *P* = 0.031; total *R^2^* = 0.137].


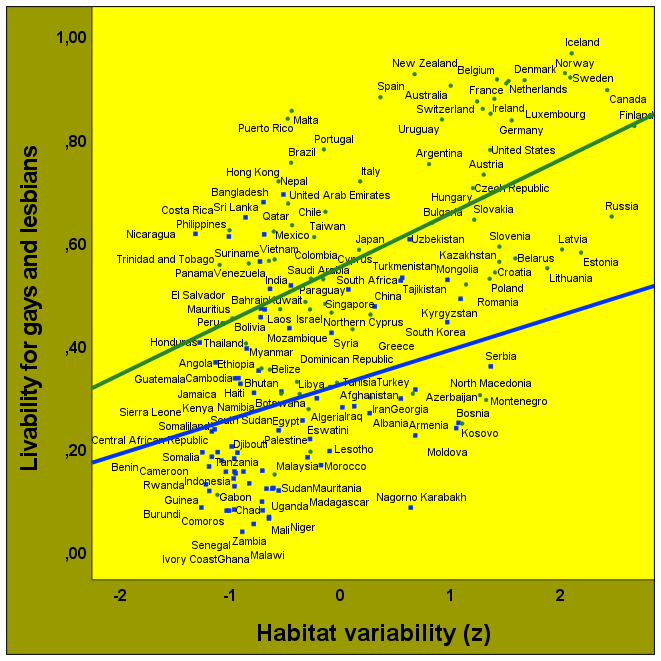


**S2 Fig | Effects of habitat variability on perceived livability for gays and lesbians in poor (blue) and rich (green) societies.** Perceived local livability in more variable habitats: *r*_(161)_ = 0.588; *P* < 0.001; CI = 0.478 to 0.681. The increases are larger for richer societies [*r*_(77)_ = 0.490; *P* < 0.001; CI = 0.301 to 0.641] than for poorer societies [*r*_(82)_ = 0.261; *P* = 0.017; CI = 0.049 to 0.450; *z* = 1.684; *P* = 0.046; total *R^2^* = 0.632].


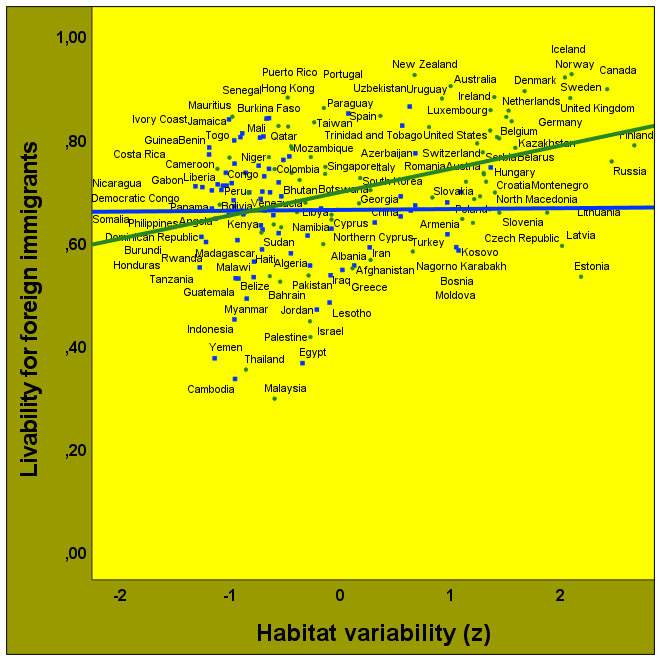


**S3 Fig | Effects of habitat variability on perceived livability for foreign immigrants in poor (blue) and rich (green) societies.** Perceived local livability increases in more variable habitats: *r*_(161)_ = 0.306; *P* < 0.001; CI = 0.160 to 0.439. The increases are larger for richer societies [*r*_(77)_ = 0.353; *P* < 0.001; CI = 0.143 to 0.533] than for poorer societies [*r*_(82)_ = 0.010; *P* = 0.928; CI = -0.205 to 0.224; *z* = 2.247; *P* = 0.012; total *R^2^* = 0.181].

**
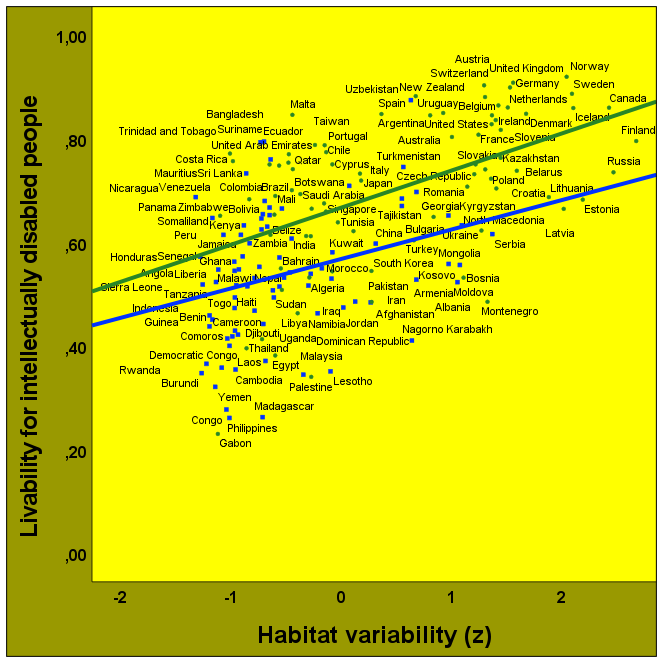
**

**S4 Fig | Effects of habitat variability on perceived livability for intellectually disabled people in poor (blue) and rich (green) societies.** Perceived local livability increases in more variable habitats: *r*_(161)_ = 0.576; *P* < 0.001; CI = 0.464 to 0.671. The increases are larger for richer societies [*r*_(77)_ = 0.507; *P* < 0.001; CI = 0.322 to 0.655] than for poorer societies [*r*_(82)_ = 0.305; *P* = 0.005; CI = 0.097 to 0.488; *z* = 1.526; *P* = 0.064; total *R^2^* = 0.484].

**S1 Table | Supplementary data for the descriptive geographical model.**

|  |  |  |  |  |  |  |  |  |  |
| --- | --- | --- | --- | --- | --- | --- | --- | --- | --- |
| Country | *N*^a^ | Latitude^b^ | Longitude | Perceived livability for minorities^c^ | | | | | Perceived livability for oneself^d^ |
|  |  |  |  |  |  |  |  |  |  |
|  |  |  |  | Racial and ethnic minorities | Gay or lesbian people | Foreign immi-grants | Intellec-tually disabled | Average perceived  livability |  |
|  |  |  |  |  |  |  |  |  |  |
| Afghanistan | 6724 | 33.94 | 67.71 | .71 | .28 | .55 | .49 | .51 | 74.45 |
| Albania | 5996 | 41.15 | 20.17 | .65 | .27 | .59 | .48 | .50 | 63.81 |
| Algeria | 5951 | 28.03 | 1.66 | .30 | .28 | .53 | .53 | .41 | 77.86 |
| Angola | 3932 | -11.20 | 17.87 | .57 | .37 | .64 | .52 | .53 | 53.41 |
| Argentina | 5728 | -38.42 | -63.62 | .80 | .75 | .82 | .85 | .81 | 81.60 |
| Armenia | 5529 | 40.07 | 45.04 | .67 | .22 | .67 | .53 | .52 | 53.77 |
| Australia | 4418 | -25.27 | 133.78 | .90 | .90 | .90 | .80 | .88 | 91.61 |
| Austria | 6399 | 47.52 | 14.55 | .76 | .73 | .73 | .88 | .78 | 93.66 |
| Azerbaijan | 5792 | 40.14 | 47.58 | .74 | .31 | .77 | .70 | .63 | 74.76 |
| Bahrain | 6206 | 25.93 | 50.64 | .61 | .47 | .52 | .55 | .54 | 87.15 |
| Bangladesh | 6958 | 23.68 | 90.36 | .89 | .68 | .80 | .79 | .79 | 90.23 |
| Belarus | 5658 | 53.71 | 27.95 | .77 | .57 | .78 | .74 | .71 | 78.79 |
| Belgium | 5278 | 50.50 | 4.47 | .81 | .91 | .80 | .86 | .85 | 88.18 |
| Belize | 392 | 17.19 | -88.50 | .76 | .35 | .53 | .61 | .56 | 72.45 |
| Benin | 4818 | 9.31 | 2.32 | .77 | .16 | .77 | .44 | .53 | 56.85 |
| Bhutan | 3014 | 27.51 | 90.43 | .76 | .31 | .69 | .67 | .60 | 93.43 |
| Bolivia | 5965 | -16.29 | -63.59 | .70 | .45 | .68 | .65 | .62 | 83.55 |
| Bosnia | 5746 | 43.92 | 17.68 | .59 | .25 | .64 | .53 | .50 | 65.52 |
| Botswana | 5858 | -22.33 | 24.68 | .72 | .31 | .72 | .69 | .61 | 60.04 |
| Brazil | 7070 | -14.24 | -51.93 | .84 | .75 | .78 | .70 | .77 | 72.99 |
| Bulgaria | 6925 | 42.73 | 25.49 | .76 | .64 | .69 | .65 | .68 | 75.58 |
| Burkina Faso | 4560 | 12.24 | -1.56 | .86 | .12 | .84 | .63 | .61 | 63.99 |
| Burundi | 1973 | -3.37 | 29.92 | .74 | .08 | .61 | .35 | .44 | 59.25 |
| Cambodia | 5990 | 12.57 | 104.99 | .62 | .34 | .33 | .35 | .41 | 89.42 |
| Cameroon | 6101 | 7.37 | 12.35 | .76 | .13 | .75 | .43 | .52 | 59.97 |
| Canada | 4924 | 56.13 | -106.35 | .91 | .89 | .90 | .86 | .89 | 90.29 |
| Central African Republic | 1980 | 6.61 | 20.94 | .70 | .20 | .71 | .42 | .51 | 71.16 |
| Chad | 5791 | 15.45 | 18.73 | .68 | .08 | .62 | .44 | .46 | 66.21 |
| Chile | 5992 | -35.68 | -71.54 | .75 | .66 | .74 | .77 | .73 | 81.89 |
| China | 27859 | 35.86 | 104.20 | .64 | .47 | .64 | .60 | .59 | 78.06 |
| Colombia | 5972 | 4.57 | -74.30 | .76 | .57 | .74 | .69 | .69 | 81.88 |
| Comoros | 4873 | -11.88 | 43.87 | .60 | .08 | .71 | .41 | .45 | 75.28 |
| Congo | 4724 | -0.23 | 15.83 | .68 | .15 | .73 | .28 | .46 | 62.17 |
| Costa Rica | 5527 | 9.75 | -83.75 | .81 | .62 | .76 | .77 | .74 | 86.67 |
| Croatia | 5619 | 45.10 | 15.20 | .72 | .54 | .70 | .70 | .66 | 53.70 |
| Cyprus | 4526 | 35.13 | 33.43 | .72 | .54 | .64 | .75 | .66 | 81.20 |
| Czech Republic | 6887 | 49.82 | 15.47 | .63 | .70 | .64 | .73 | .68 | 70.71 |
| Democratic Congo | 4873 | -4.04 | 21.76 | .66 | .18 | .70 | .36 | .47 | 86.88 |
| Denmark | 5497 | 56.26 | 9.50 | .88 | .91 | .89 | .85 | .88 | 93.42 |
| Djibouti | 1821 | 11.83 | 42.59 | .63 | .19 | .60 | .42 | .46 | 75.49 |
| Dominican Republic | 5872 | 18.74 | -70.16 | .63 | .35 | .62 | .41 | .50 | 75.20 |
| Ecuador | 5938 | -1.83 | -78.18 | .76 | .51 | .70 | .76 | .68 | 81.91 |
| Egypt | 14632 | 26.82 | 30.80 | .36 | .25 | .36 | .34 | .33 | 69.81 |
| El Salvador | 5977 | 13.79 | -88.90 | .66 | .47 | .63 | .63 | .60 | 82.30 |
| Estonia | 4253 | 58.60 | 25.01 | .61 | .58 | .53 | .68 | .60 | 85.73 |
| Eswatini | 979 | -26.52 | 31.47 | .43 | .22 | .55 | .54 | .43 | 67.42 |
| Ethiopia | 4991 | 9.15 | 40.49 | .79 | .35 | .75 | .55 | .61 | 73.11 |
| Finland | 5627 | 61.92 | 25.75 | .78 | .82 | .79 | .80 | .80 | 91.24 |
| France | 6221 | 46.23 | 2.21 | .82 | .87 | .79 | .81 | .82 | 87.23 |
| Gabon | 4591 | -0.80 | 11.61 | .76 | .11 | .74 | .23 | .46 | 47.53 |
| Georgia | 5910 | 42.32 | 43.36 | .78 | .30 | .69 | .68 | .61 | 76.80 |
| Germany | 17304 | 51.17 | 10.45 | .86 | .84 | .83 | .91 | .86 | 93.82 |
| Ghana | 5965 | 7.95 | -1.02 | .59 | .08 | .67 | .55 | .47 | 57.05 |
| Greece | 5319 | 39.07 | 21.82 | .52 | .43 | .55 | .62 | .53 | 78.81 |
| Guatemala | 5970 | 15.78 | -90.23 | .74 | .34 | .53 | .55 | .54 | 86.63 |
| Guinea | 4818 | 9.95 | -9.70 | .74 | .12 | .78 | .46 | .52 | 63.03 |
| Haiti | 2997 | 18.97 | -72.29 | .41 | .31 | .56 | .47 | .44 | 44.18 |
| Honduras | 5968 | 15.20 | -86.24 | .66 | .40 | .55 | .57 | .55 | 82.67 |
| Hong Kong | 2689 | 22.40 | 114.11 | .72 | .72 | .82 | .75 | .75 | 83.04 |
| Hungary | 5901 | 47.16 | 19.50 | .73 | .69 | .73 | .74 | .72 | 77.41 |
| Iceland | 1462 | 64.96 | -19.02 | .92 | .97 | .92 | .86 | .92 | 79.41 |
| India | 30936 | 20.59 | 78.96 | .76 | .52 | .58 | .61 | .62 | 77.35 |
| Indonesia | 7063 | -0.79 | 113.92 | .72 | .15 | .45 | .49 | .46 | 88.06 |
| Iran | 8480 | 32.43 | 53.69 | .68 | .30 | .56 | .49 | .51 | 73.68 |
| Iraq | 9951 | 33.22 | 43.68 | .52 | .28 | .55 | .47 | .46 | 61.63 |
| Ireland | 5075 | 53.41 | -8.24 | .90 | .88 | .88 | .84 | .87 | 91.59 |
| Israel | 4384 | 31.05 | 34.85 | .46 | .47 | .45 | .61 | .50 | 80.61 |
| Italy | 6690 | 41.87 | 12.57 | .75 | .72 | .72 | .72 | .73 | 74.29 |
| Ivory Coast | 2830 | 7.54 | -5.55 | .82 | .08 | .84 | .40 | .53 | 50.71 |
| Jamaica | 1495 | 18.11 | -77.30 | .77 | .32 | .80 | .61 | .63 | 63.88 |
| Japan | 5963 | 36.20 | 138.25 | .70 | .58 | .67 | .73 | .67 | 83.29 |
| Jordan | 7751 | 30.59 | 36.24 | .53 | .30 | .47 | .46 | .44 | 75.02 |
| Kazakhstan | 5443 | 48.02 | 66.92 | .82 | .56 | .80 | .76 | .74 | 77.99 |
| Kenya | 5943 | -0.02 | 37.91 | .70 | .15 | .65 | .63 | .54 | 64.88 |
| Kosovo | 5907 | 42.60 | 20.90 | .63 | .25 | .58 | .56 | .50 | 69.56 |
| Kuwait | 4749 | 29.31 | 47.48 | .65 | .48 | .68 | .61 | .61 | 86.10 |
| Kyrgyzstan | 5700 | 41.20 | 74.77 | .77 | .44 | .68 | .65 | .64 | 84.86 |
| Laos | 1991 | 19.86 | 102.50 | .82 | .47 | .66 | .37 | .58 | 94.67 |
| Latvia | 4307 | 56.88 | 24.60 | .71 | .59 | .59 | .66 | .64 | 86.21 |
| Lebanon | 8791 | 33.85 | 35.86 | .47 | .18 | .61 | .52 | .44 | 76.47 |
| Lesotho | 994 | -29.61 | 28.23 | .36 | .19 | .48 | .35 | .35 | 53.52 |
| Liberia | 4847 | 6.43 | -9.43 | .50 | .19 | .71 | .55 | .49 | 55.25 |
| Libya | 1897 | 26.34 | 17.23 | .61 | .33 | .66 | .46 | .51 | 75.12 |
| Lithuania | 5777 | 55.17 | 23.88 | .69 | .55 | .66 | .69 | .65 | 82.55 |
| Luxembourg | 3729 | 49.82 | 6.13 | .87 | .85 | .85 | .85 | .85 | 93.98 |
| Madagascar | 5011 | -18.77 | 46.87 | .73 | .10 | .59 | .26 | .42 | 75.45 |
| Malawi | 4891 | -13.25 | 34.30 | .62 | .05 | .53 | .53 | .43 | 73.67 |
| Malaysia | 5887 | 4.21 | 101.98 | .70 | .15 | .30 | .38 | .38 | 81.83 |
| Mali | 5865 | 17.57 | -4.00 | .87 | .06 | .84 | .67 | .61 | 61.11 |
| Malta | 5228 | 35.94 | 14.38 | .77 | .85 | .73 | .85 | .80 | 83.78 |
| Mauritania | 7642 | 21.01 | -10.94 | .55 | .12 | .65 | .49 | .45 | 65.12 |
| Mauritius | 1985 | -20.35 | 57.55 | .86 | .45 | .84 | .76 | .73 | 90.88 |
| Mexico | 6942 | 23.63 | -102.55 | .65 | .62 | .63 | .65 | .64 | 74.63 |
| Moldova | 5808 | 47.41 | 28.37 | .57 | .24 | .59 | .52 | .48 | 72.12 |
| Mongolia | 5990 | 46.86 | 103.85 | .69 | .53 | .61 | .56 | .60 | 77.85 |
| Montenegro | 5476 | 42.71 | 19.37 | .72 | .29 | .72 | .49 | .55 | 67.91 |
| Morocco | 8027 | 31.79 | -7.09 | .65 | .17 | .66 | .55 | .51 | 73.34 |
| Mozambique | 1982 | -18.67 | 35.53 | .67 | .43 | .77 | .54 | .60 | 81.18 |
| Myanmar | 4078 | 21.91 | 95.96 | .76 | .39 | .49 | .52 | .54 | 90.73 |
| Nagorno Karabakh | 889 | 39.80 | 46.66 | .64 | .08 | .66 | .41 | .45 | 67.27 |
| Namibia | 946 | -22.96 | 18.49 | .62 | .31 | .63 | .51 | .52 | 64.59 |
| Nepal | 6875 | 28.39 | 84.12 | .85 | .69 | .76 | .53 | .71 | 86.28 |
| Netherlands | 5299 | 52.13 | 5.29 | .84 | .91 | .85 | .90 | .88 | 93.01 |
| New Zealand | 3943 | -40.90 | 174.89 | .91 | .92 | .92 | .88 | .91 | 89.25 |
| Nicaragua | 5980 | 12.87 | -85.21 | .77 | .62 | .71 | .69 | .70 | 86.17 |
| Niger | 5921 | 17.61 | 8.08 | .83 | .07 | .74 | .65 | .57 | 74.09 |
| Nigeria | 6932 | 9.08 | 8.68 | .62 | .14 | .68 | .56 | .50 | 60.86 |
| North Macedonia | 5868 | 41.61 | 21.75 | .66 | .30 | .69 | .62 | .57 | 66.36 |
| Northern Cyprus | 2051 | 35.25 | 33.66 | .77 | .46 | .65 | .54 | .61 | 83.17 |
| Norway | 2771 | 60.47 | 8.47 | .90 | .93 | .92 | .92 | .92 | 93.88 |
| Pakistan | 6873 | 30.38 | 69.35 | .74 | .32 | .54 | .53 | .53 | 81.54 |
| Palestine | 8369 | 31.65 | 34.77 | .30 | .19 | .42 | .34 | .31 | 73.69 |
| Panama | 5773 | 8.54 | -80.78 | .78 | .55 | .67 | .65 | .66 | 84.20 |
| Paraguay | 5825 | -23.44 | -58.44 | .66 | .51 | .85 | .71 | .68 | 88.43 |
| Peru | 5990 | -9.19 | -75.02 | .68 | .44 | .71 | .61 | .61 | 72.27 |
| Philippines | 6998 | 12.88 | 121.77 | .54 | .61 | .65 | .26 | .51 | 86.67 |
| Poland | 6942 | 51.92 | 19.15 | .68 | .53 | .66 | .72 | .65 | 87.87 |
| Portugal | 6517 | 39.40 | -8.22 | .83 | .78 | .86 | .79 | .81 | 87.58 |
| Puerto Rico | 464 | 18.22 | -66.59 | .89 | .84 | .88 | .75 | .84 | 84.70 |
| Qatar | 1645 | 25.35 | 51.18 | .78 | .63 | .78 | .74 | .73 | 93.04 |
| Romania | 5978 | 45.94 | 24.97 | .77 | .52 | .72 | .71 | .68 | 80.03 |
| Russia | 14120 | 61.52 | 105.32 | .75 | .65 | .76 | .74 | .72 | 73.75 |
| Rwanda | 4712 | -1.94 | 29.87 | .65 | .13 | .60 | .37 | .44 | 68.15 |
| Saudi Arabia | 7952 | 23.89 | 45.08 | .76 | .53 | .76 | .67 | .68 | 86.46 |
| Senegal | 4893 | 14.50 | -14.45 | .89 | .04 | .81 | .57 | .58 | 55.53 |
| Serbia | 5498 | 44.02 | 21.01 | .78 | .36 | .74 | .62 | .62 | 62.31 |
| Sierra Leone | 4946 | 8.46 | -11.78 | .53 | .19 | .71 | .52 | .49 | 57.60 |
| Singapore | 3983 | 1.35 | 103.82 | .90 | .48 | .73 | .66 | .69 | 92.52 |
| Slovakia | 5930 | 48.67 | 19.70 | .70 | .64 | .68 | .75 | .69 | 84.52 |
| Slovenia | 5583 | 46.15 | 15.00 | .76 | .59 | .66 | .82 | .71 | 89.87 |
| Somalia | 1942 | 5.15 | 46.20 | .74 | .18 | .66 | .45 | .51 | 87.23 |
| Somaliland | 4756 | 5.15 | 46.20 | .77 | .23 | .70 | .65 | .59 | 81.38 |
| South Africa | 6800 | -30.56 | 22.94 | .67 | .53 | .60 | .68 | .62 | 56.10 |
| South Korea | 5952 | 35.91 | 127.77 | .74 | .46 | .70 | .55 | .61 | 76.67 |
| South Sudan | 1860 | 6.88 | 31.31 | .59 | .23 | .72 | .57 | .53 | 51.51 |
| Spain | 6401 | 40.46 | -3.75 | .82 | .88 | .84 | .85 | .85 | 85.84 |
| Sri Lanka | 7203 | 7.87 | 80.77 | .84 | .65 | .73 | .73 | .74 | 89.16 |
| Sudan | 5705 | 12.86 | 30.22 | .64 | .12 | .62 | .51 | .47 | 62.60 |
| Suriname | 504 | 3.92 | -56.03 | .74 | .56 | .80 | .79 | .72 | 89.48 |
| Sweden | 5286 | 60.13 | 18.64 | .88 | .92 | .88 | .89 | .89 | 93.38 |
| Switzerland | 2053 | 46.82 | 8.23 | .79 | .86 | .77 | .90 | .83 | 95.22 |
| Syria | 7801 | 34.80 | 39.00 | .64 | .42 | .62 | .58 | .57 | 42.89 |
| Taiwan | 5825 | 23.70 | 120.96 | .83 | .61 | .83 | .79 | .77 | 81.83 |
| Tajikistan | 5952 | 38.86 | 71.28 | .83 | .52 | .65 | .67 | .67 | 87.99 |
| Tanzania | 5984 | -6.37 | 34.89 | .62 | .15 | .53 | .52 | .45 | 62.92 |
| Thailand | 6985 | 15.87 | 100.99 | .40 | .40 | .35 | .40 | .39 | 93.50 |
| Togo | 2877 | 8.62 | 0.82 | .81 | .18 | .80 | .47 | .57 | 48.07 |
| Trinidad and Tobago | 979 | 10.69 | -61.22 | .79 | .56 | .76 | .75 | .72 | 79.98 |
| Tunisia | 9223 | 33.89 | 9.54 | .52 | .33 | .66 | .64 | .54 | 69.72 |
| Turkey | 7887 | 38.96 | 35.24 | .55 | .31 | .58 | .60 | .51 | 77.10 |
| Turkmenistan | 4907 | 38.97 | 59.56 | .78 | .53 | .82 | .75 | .72 | 93.48 |
| Uganda | 5918 | 1.37 | 32.29 | .66 | .12 | .67 | .51 | .49 | 62.66 |
| Ukraine | 5658 | 48.38 | 31.17 | .69 | .49 | .70 | .63 | .63 | 75.22 |
| United Arab Emirates | 5483 | 23.42 | 53.85 | .81 | .67 | .82 | .77 | .77 | 93.22 |
| United Kingdom | 15822 | 55.38 | -3.44 | .89 | .91 | .84 | .86 | .88 | 88.54 |
| United States | 5504 | 37.09 | -95.71 | .85 | .78 | .82 | .83 | .82 | 83.79 |
| Uruguay | 5823 | -32.52 | -55.77 | .87 | .84 | .88 | .85 | .86 | 79.68 |
| Uzbekistan | 5818 | 41.38 | 64.59 | .91 | .60 | .86 | .88 | .81 | 93.38 |
| Venezuela | 5860 | 6.42 | -66.59 | .64 | .56 | .70 | .68 | .65 | 78.23 |
| Vietnam | 7006 | 14.06 | 108.28 | .74 | .61 | .73 | .68 | .69 | 82.14 |
| Yemen | 8911 | 15.55 | 48.52 | .42 | .24 | .37 | .32 | .34 | 69.80 |
| Zambia | 4931 | -13.13 | 27.85 | .63 | .13 | .70 | .60 | .51 | 56.50 |
| Zimbabwe | 5865 | -19.02 | 29.15 | .65 | .16 | .70 | .65 | .54 | 64.71 |
|  |  |  |  |  |  |  |  |  |  |
| ^a^ Number of native respondents of the Gallup World Polls from 2010 to 2015.  ^b^ Midrange latitude and midrange longitude retrieved from [https://developers.google.­com/public-data/docs/canonical/countries_csv](https://developers.google.com/public-data/docs/canonical/countries_csv).  ^c^ Country-level proportions of *a good place to live* for racial and ethnic minorities, gay or lesbian people, immigrants from other countries, and intellectually disabled people, retrieved from <https://www.gallup.com/analytics/318923/world-poll-public-datasets.aspx>.  ^d^ Country-level percentages of *respondents satisfied with the city or area where they live*, retrieved from https://www.gallup.com/analytics/318923/world-poll-public-datasets.aspx. | | | | | | | | | |
|  |  |  |  |  |  |  |  |  |  |

**S2 Table | Descriptive effects of latitude and longitude on perceived livability for minorities (total *N* = 163), broken down by hemisphere.**

|  |  |  |  | | |  |  |
| --- | --- | --- | --- | --- | --- | --- | --- |
| Global area | Hemisphere | | | | | | Entire world |
|  |  |  |  | | |  |  |
|  | North | South | East | | | West |  |
|  |  |  | |  |  | |  |
| Number of countries | 132 | 31 | | 122 | 41 | | 163 |
|  |  |  | |  |  | |  |
| Latitude | 0.004^***^  (0.003 to 0.005) | -0.007^***^  (-0.011 to -0.003) | | 0.084^***^  (0.062 to 0.107) | 0.089^***^  (0.052 to 0.125) | | 0.070^***^  (0.050 to 0.090) |
| Squared latitude | Not applicable | Not applicable | | 0.059^***^  (0.039 to 0.079) | 0.070^***^  (0.048 to 0.092) | | 0.058^***^  (0.042 to 0.074) |
| Longitude | -0.001  (-0.001 to 0.000) | 0.000  (-0.001 to 0.001) | | 0.046^**^  (0.014 to 0.078) | -0.045  (-0.093 to 0.003) | | -0.023^*^  (-0.041 to -0.004) |
|  |  |  | |  |  | |  |
| *R^2^* | 0.272^***^ (0.233^a^) | 0.346^***^ (0.334^a^) | | 0.369^***^ (0.326^a^) | 0.559^***^ (0.516^a^) | | 0.330^***^ (0.304^a^) |
|  |  |  |  | | |  |  |
| Shown are unstandardized regression coefficients (*B*) with 95% confidence intervals between brackets. ^*^*P* < 0.05. ^**^*P* < 0.01. ^***^*P* < 0.001.  ^a^Before controlling for longitude. | | | | | | | |

**S3 Table | Descriptive effects of latitude and longitude on perceived livability for minorities (*N* = 163), broken down by minority group.**

|  |  |  |  |  |  |
| --- | --- | --- | --- | --- | --- |
| Predictor | Perceived livability for minorities | | | | |
|  |  |  |  |  |  |
|  | Racial and ethnic minorities | Gay or lesbian people | Immigrants from other countries | Intellectually disabled people | Overall  livability |
|  |  |  |  |  |  |
| Latitude (z) | 0.028^**^  (0.008, 0.049) | 0.141^***^  (0.108, 0.175) | 0.028^**^  (0.009, 0.047) | 0.082^***^  (0.061, 0.104) | 0.070^***^  (0.050, 0.090) |
| Latitude-squared | 0.025^**^  (0.008, 0.041) | 0.106^***^  (0.079, 0.134) | 0.035^***^  (0.020, 0.051) | 0.065^***^  (0.048, 0.083) | 0.059^***^  (0.042, 0.074) |
| Longitude (z) | -0.006  (-0.026, 0.013) | -0.028  (-0.059, 0.003) | -0.024^**^  (-0.042, -0.006) | -0.032^**^  (-0.052, -0.012) | -0.023^*^  (-0.041, -0.004) |
|  |  |  |  |  |  |
| *R^2^* | 0.069^***^ | 0.377^***^ | 0.149^***^ | 0.364^***^ | 0.330^***^ |
|  |  |  |  |  |  |
| Shown are unstandardized regression coefficients (*B*) with 95% confidence intervals between brackets. ^*^*P* < 0.05. ^**^*P* < 0.01. ^***^*P* < 0.001. | | | | | |

**S4 Table | Supplementary data for the explanatory ecological model.**

|  |  |  |  |  | |  |  |  |
| --- | --- | --- | --- | --- | --- | --- | --- | --- |
| Country | Abiotic habitat variability | | | | | National wealth^e^ | Mediators | |
|  |  |  |  |  | |  |  |  |
|  | Day-length variability^a^ | Temperature variability^b^ | Precipitation variability^c^ | | Habitat variability^d^ |  | Governmental quality^f^ | Psychosocial flourishing^g^ |
|  |  |  |  |  | |  |  |  |
| Afghanistan | 4.57 | 21.64 | 0.00 | 0.25 | | 6.81 | -1.68 | 0.54 |
| Albania | 6.02 | 17.21 | 0.15 | 0.40 | | 8.40 | 0.02 | 0.55 |
| Algeria | 3.59 | 17.46 | 0.00 | -0.18 | | 8.68 | -0.85 | 0.61 |
| Angola | 1.79 | 8.51 | 0.00 | -1.05 | | 7.68 | -0.98 | 0.56 |
| Argentina | 5.43 | 17.61 | 0.51 | 0.95 | | 9.41 | 0.03 | 0.66 |
| Armenia | 5.78 | 23.81 | 0.15 | 0.82 | | 8.09 | -0.11 | 0.45 |
| Australia | 3.18 | 19.70 | 0.73 | 1.16 | | 10.24 | 1.77 | 0.77 |
| Austria | 7.69 | 21.68 | 0.46 | 1.47 | | 10.29 | 1.64 | 0.72 |
| Azerbaijan | 5.79 | 23.81 | 0.15 | 0.82 | | 8.14 | -0.70 | 0.55 |
| Bahrain | 3.27 | 14.27 | 0.00 | -0.44 | | 9.78 | -0.18 | 0.68 |
| Bangladesh | 2.95 | 12.54 | 0.00 | -0.60 | | 7.45 | -0.85 | 0.56 |
| Belarus | 9.99 | 22.72 | 0.41 | 1.76 | | 8.81 | -0.52 | 0.53 |
| Belgium | 8.68 | 19.25 | 0.56 | 1.59 | | 10.26 | 1.33 | 0.70 |
| Belize | 2.07 | 10.83 | 0.18 | -0.54 | | 8.72 | -0.31 | 0.65 |
| Benin | 1.78 | 6.80 | 0.04 | -1.11 | | 6.96 | -0.32 | 0.56 |
| Bhutan | 3.51 | 13.75 | 0.01 | -0.43 | | 7.43 | 0.65 | 0.68 |
| Bolivia | 1.96 | 12.56 | 0.07 | -0.63 | | 7.84 | -0.58 | 0.69 |
| Bosnia | 6.69 | 22.83 | 0.38 | 1.27 | | 8.78 | -0.38 | 0.46 |
| Botswana | 2.76 | 17.89 | 0.00 | -0.26 | | 9.04 | 0.65 | 0.69 |
| Brazil | 1.82 | 11.39 | 0.30 | -0.34 | | 8.97 | -0.24 | 0.66 |
| Bulgaria | 6.39 | 20.80 | 0.32 | 0.98 | | 8.85 | 0.28 | 0.48 |
| Burkina Faso | 1.80 | 15.34 | 0.00 | -0.57 | | 6.99 | -0.44 | 0.60 |
| Burundi | 1.75 | 5.70 | 0.04 | -1.19 | | 6.45 | -1.53 | 0.48 |
| Cambodia | 1.80 | 10.45 | 0.03 | -0.87 | | 7.59 | -0.83 | 0.60 |
| Cameroon | 1.76 | 9.23 | 0.08 | -0.87 | | 7.58 | -1.11 | 0.67 |
| Canada | 11.26 | 27.25 | 0.63 | 2.63 | | 10.29 | 1.78 | 0.77 |
| Central African Republic | 1.77 | 10.20 | 0.02 | -0.90 | | 7.04 | -1.68 | 0.57 |
| Chad | 1.85 | 14.72 | 0.00 | -0.61 | | 7.19 | -1.47 | 0.58 |
| Chile | 4.89 | 16.29 | 0.04 | -0.02 | | 9.21 | 1.14 | 0.68 |
| China | 4.92 | 23.43 | 0.01 | 0.44 | | 8.48 | -0.29 | 0.56 |
| Colombia | 1.75 | 8.84 | 0.32 | -0.50 | | 8.80 | -0.17 | 0.73 |
| Comoros | 1.79 | 5.70 | 0.18 | -0.95 | | 7.46 | -0.96 | 0.59 |
| Congo | 1.75 | 9.97 | 0.00 | -0.95 | | 6.83 | -0.70 | 0.61 |
| Costa Rica | 1.78 | 10.01 | 0.02 | -0.92 | | 9.10 | -1.77 | 0.74 |
| Croatia | 6.99 | 24.22 | 0.48 | 1.57 | | 9.23 | 0.66 | 0.47 |
| Cyprus | 4.79 | 17.85 | 0.01 | 0.04 | | 9.93 | 0.50 | 0.64 |
| Czech Republic | 8.44 | 23.57 | 0.26 | 1.37 | | 9.78 | 0.92 | 0.55 |
| Democratic Congo | 1.75 | 9.04 | 0.01 | -1.00 | | 6.56 | -0.25 | 0.56 |
| Denmark | 11.34 | 20.29 | 0.45 | 1.85 | | 10.31 | 1.06 | 0.73 |
| Djibouti | 1.80 | 11.43 | 0.00 | -0.85 | | 7.60 | 1.88 | 0.58 |
| Dominican Republic | 2.27 | 9.04 | 0.19 | -0.62 | | 8.81 | -0.84 | 0.72 |
| Ecuador | 1.75 | 13.15 | 0.11 | -0.54 | | 8.18 | -0.42 | 0.66 |
| Egypt | 3.41 | 17.00 | 0.00 | -0.23 | | 8.26 | -0.84 | 0.55 |
| El Salvador | 1.82 | 14.18 | 0.02 | -0.63 | | 8.48 | -0.38 | 0.65 |
| Estonia | 13.02 | 23.54 | 0.49 | 2.38 | | 9.42 | 1.37 | 0.56 |
| Eswatini | 3.37 | 16.19 | 0.08 | -0.16 | | 8.50 | -0.57 | 0.69 |
| Ethiopia | 1.77 | 13.95 | 0.02 | -0.65 | | 6.60 | -0.86 | 0.58 |
| Finland | 16.64 | 23.54 | 0.49 | 2.88 | | 10.21 | 1.97 | 0.73 |
| France | 7.31 | 19.56 | 0.55 | 1.41 | | 10.20 | 1.27 | 0.69 |
| Gabon | 1.75 | 8.65 | 0.01 | -1.03 | | 8.78 | -0.79 | 0.61 |
| Georgia | 6.29 | 19.69 | 0.20 | 0.69 | | 7.86 | 0.52 | 0.48 |
| Germany | 8.94 | 22.11 | 0.50 | 1.73 | | 10.20 | 1.68 | 0.74 |
| Ghana | 1.77 | 9.01 | 0.08 | -0.88 | | 7.66 | 0.07 | 0.69 |
| Greece | 5.57 | 17.41 | 0.08 | 0.24 | | 9.86 | 0.32 | 0.50 |
| Guatemala | 1.89 | 11.12 | 0.01 | -0.84 | | 8.31 | -0.64 | 0.73 |
| Guinea | 1.79 | 7.60 | 0.00 | -1.11 | | 7.64 | -1.00 | 0.63 |
| Haiti | 2.30 | 9.22 | 0.14 | -0.69 | | 7.41 | -1.23 | 0.52 |
| Honduras | 1.83 | 6.18 | 0.01 | -1.20 | | 7.88 | -0.67 | 0.67 |
| Hong Kong | 2.76 | 13.15 | 0.08 | -0.46 | | 10.23 | 1.66 | 0.64 |
| Hungary | 7.58 | 22.07 | 0.46 | 1.47 | | 9.56 | 0.53 | 0.51 |
| Iceland | 17.49 | 14.55 | 0.45 | 2.29 | | 10.34 | 1.72 | 0.73 |
| India | 2.52 | 16.80 | 0.02 | -0.34 | | 7.91 | -0.08 | 0.55 |
| Indonesia | 1.75 | 7.66 | 0.14 | -0.87 | | 8.10 | -0.11 | 0.69 |
| Iran | 4.30 | 22.82 | 0.07 | 0.40 | | 8.81 | -1.05 | 0.63 |
| Iraq | 4.44 | 20.31 | 0.00 | 0.14 | | 8.43 | -1.64 | 0.55 |
| Ireland | 9.86 | 15.35 | 0.61 | 1.57 | | 10.46 | 1.56 | 0.75 |
| Israel | 4.08 | 16.68 | 0.00 | -0.16 | | 9.97 | 0.78 | 0.60 |
| Italy | 6.18 | 16.47 | 0.12 | 0.31 | | 10.17 | 0.55 | 0.60 |
| Ivory Coast | 1.76 | 8.38 | 0.08 | -0.92 | | 7.36 | -0.51 | 0.63 |
| Jamaica | 2.18 | 9.07 | 0.08 | -0.82 | | 8.28 | 0.28 | 0.71 |
| Japan | 4.99 | 16.52 | 0.21 | 0.30 | | 10.23 | 1.50 | 0.59 |
| Jordan | 4.00 | 17.76 | 0.00 | -0.10 | | 8.36 | -0.04 | 0.61 |
| Kazakhstan | 7.85 | 26.62 | 0.33 | 1.61 | | 8.76 | -0.32 | 0.59 |
| Kenya | 1.75 | 10.62 | 0.07 | -0.79 | | 6.97 | -0.58 | 0.70 |
| Kosovo | 6.49 | 23.03 | 0.35 | 1.23 | | 8.61 | -0.39 | 0.55 |
| Kuwait | 3.79 | 16.63 | 0.00 | -0.21 | | 9.75 | -0.09 | 0.77 |
| Kyrgyzstan | 6.04 | 25.79 | 0.23 | 1.12 | | 7.64 | -0.67 | 0.60 |
| Laos | 2.42 | 13.65 | 0.01 | -0.59 | | 7.47 | -0.82 | 0.58 |
| Latvia | 11.73 | 23.54 | 0.49 | 2.20 | | 9.14 | 0.90 | 0.54 |
| Lebanon | 4.56 | 15.41 | 0.00 | -0.19 | | 8.48 | -0.87 | 0.57 |
| Lesotho | 3.84 | 17.67 | 0.09 | 0.02 | | 7.77 | -0.33 | 0.67 |
| Liberia | 1.77 | 8.09 | 0.03 | -1.03 | | 6.45 | -0.79 | 0.72 |
| Libya | 3.34 | 16.39 | 0.00 | -0.29 | | 8.93 | -2.04 | 0.71 |
| Lithuania | 10.72 | 23.54 | 0.49 | 2.06 | | 9.23 | 0.99 | 0.45 |
| Luxembourg | 8.44 | 20.27 | 0.50 | 1.53 | | 11.01 | 1.93 | 0.71 |
| Madagascar | 2.27 | 13.16 | 0.03 | -0.62 | | 6.70 | -0.81 | 0.55 |
| Malawi | 1.81 | 13.61 | 0.00 | -0.69 | | 6.42 | -0.51 | 0.65 |
| Malaysia | 1.75 | 8.35 | 0.34 | -0.50 | | 9.16 | 0.56 | 0.66 |
| Mali | 2.12 | 15.04 | 0.00 | -0.55 | | 6.81 | -0.94 | 0.65 |
| Malta | 4.94 | 12.52 | 0.00 | -0.34 | | 9.81 | 1.17 | 0.69 |
| Mauritania | 2.58 | 14.70 | 0.00 | -0.51 | | 7.57 | -0.80 | 0.63 |
| Mauritius | 2.49 | 9.39 | 0.00 | -0.89 | | 9.30 | 0.86 | 0.68 |
| Mexico | 2.94 | 13.35 | 0.03 | -0.50 | | 9.13 | -0.36 | 0.65 |
| Moldova | 7.66 | 21.29 | 0.33 | 1.21 | | 7.48 | -0.36 | 0.51 |
| Mongolia | 7.49 | 28.40 | 0.00 | 1.13 | | 7.52 | 0.02 | 0.58 |
| Montenegro | 6.39 | 24.22 | 0.48 | 1.49 | | 8.92 | 0.17 | 0.49 |
| Morocco | 4.20 | 17.92 | 0.00 | -0.06 | | 8.27 | -0.29 | 0.61 |
| Mozambique | 2.27 | 15.05 | 0.10 | -0.36 | | 6.95 | -0.83 | 0.62 |
| Myanmar | 2.70 | 10.78 | 0.01 | -0.76 | | 6.93 | -0.99 | 0.64 |
| Nagorno Karabakh | 5.81 | 23.81 | 0.15 | 0.78 | | 8.14 |  | 0.58 |
| Namibia | 2.85 | 15.24 | 0.00 | -0.43 | | 8.81 | 0.34 | 0.71 |
| Nepal | 3.65 | 13.75 | 0.01 | -0.41 | | 7.24 | -0.51 | 0.53 |
| Netherlands | 9.31 | 19.43 | 0.56 | 1.70 | | 10.27 | 1.90 | 0.73 |
| New Zealand | 5.97 | 12.74 | 0.59 | 0.82 | | 9.99 | 2.01 | 0.78 |
| Nicaragua | 1.80 | 5.70 | 0.00 | -1.24 | | 7.95 | -0.97 | 0.67 |
| Niger | 2.12 | 15.04 | 0.00 | -0.55 | | 6.65 | -0.76 | 0.63 |
| Nigeria | 1.78 | 9.65 | 0.05 | -0.88 | | 6.88 | -1.12 | 0.70 |
| North Macedonia | 6.13 | 22.83 | 0.52 | 1.43 | | 8.71 | -0.02 | 0.51 |
| Northern Cyprus | 4.85 | 17.85 | 0.01 | 0.04 | | 9.93 |  | 0.60 |
| Norway | 15.67 | 21.43 | 0.27 | 2.23 | | 10.46 | 1.97 | 0.76 |
| Pakistan | 3.96 | 18.57 | 0.05 | 0.03 | | 7.62 | -1.00 | 0.52 |
| Palestine | 4.10 | 16.68 | 0.00 | -0.16 | | 8.97 | -0.68 | 0.57 |
| Panama | 1.78 | 8.15 | 0.04 | -1.01 | | 8.78 | 0.12 | 0.72 |
| Paraguay | 2.92 | 18.31 | 0.24 | 0.19 | | 8.44 | -0.37 | 0.68 |
| Peru | 1.78 | 9.52 | 0.00 | -0.98 | | 8.55 | -0.13 | 0.67 |
| Philippines | 1.81 | 9.54 | 0.03 | -0.93 | | 8.36 | -0.33 | 0.75 |
| Poland | 9.22 | 22.87 | 0.32 | 1.52 | | 9.29 | 0.73 | 0.56 |
| Portugal | 5.63 | 14.82 | 0.03 | -0.03 | | 9.82 | 1.19 | 0.62 |
| Puerto Rico | 2.21 | 6.98 | 0.43 | -0.37 | | 9.85 | 0.44 | 0.71 |
| Qatar | 3.18 | 16.05 | 0.00 | -0.33 | | 9.90 | 0.43 | 0.72 |
| Romania | 7.23 | 26.23 | 0.21 | 1.30 | | 8.88 | 0.19 | 0.49 |
| Russia | 16.54 | 22.72 | 0.41 | 2.67 | | 9.09 | -0.66 | 0.53 |
| Rwanda | 1.75 | 6.38 | 0.04 | -1.14 | | 7.06 | 0.05 | 0.64 |
| Saudi Arabia | 2.97 | 18.99 | 0.00 | -0.16 | | 9.44 | -0.19 | 0.66 |
| Senegal | 1.82 | 12.03 | 0.00 | -0.80 | | 7.38 | -0.06 | 0.66 |
| Serbia | 6.71 | 24.22 | 0.48 | 1.53 | | 8.14 | -0.03 | 0.44 |
| Sierra Leone | 1.77 | 6.69 | 0.00 | -1.18 | | 6.26 | -0.63 | 0.69 |
| Singapore | 1.75 | 7.36 | 0.66 | -0.02 | | 10.13 | 1.85 | 0.61 |
| Slovakia | 8.05 | 23.44 | 0.31 | 1.38 | | 9.47 | 0.76 | 0.52 |
| Slovenia | 7.29 | 24.22 | 0.48 | 1.61 | | 9.89 | 1.06 | 0.67 |
| Somalia | 1.76 | 8.08 | 0.00 | -1.09 | | 6.66 | -2.28 | 0.65 |
| Somaliland | 1.76 | 8.08 | 0.00 | -1.09 | | 7.84 |  | 0.66 |
| South Africa | 4.00 | 16.45 | 0.09 | -0.04 | | 9.23 | 0.17 | 0.69 |
| South Korea | 4.94 | 21.82 | 0.05 | 0.40 | | 9.81 | 1.03 | 0.55 |
| South Sudan | 1.80 | 16.99 | 0.00 | -0.46 | | 7.53 | -2.26 | 0.58 |
| Spain | 5.87 | 17.60 | 0.21 | 0.50 | | 10.00 | 0.92 | 0.69 |
| Sri Lanka | 1.77 | 8.09 | 0.19 | -0.77 | | 8.25 | -0.13 | 0.74 |
| Sudan | 1.80 | 16.99 | 0.00 | -0.46 | | 7.53 | -1.68 | 0.64 |
| Suriname | 1.75 | 8.65 | 0.25 | -0.63 | | 8.64 | -0.19 | 0.66 |
| Sweden | 14.84 | 22.45 | 0.33 | 2.28 | | 10.19 | 1.90 | 0.72 |
| Switzerland | 7.48 | 21.82 | 0.47 | 1.46 | | 10.33 | 1.99 | 0.75 |
| Syria | 4.72 | 18.29 | 0.00 | 0.04 | | 8.19 | -2.11 | 0.48 |
| Taiwan | 2.95 | 14.25 | 0.22 | -0.13 | | 10.11 | 1.25 | 0.61 |
| Tajikistan | 5.52 | 24.93 | 0.05 | 0.69 | | 7.01 | -1.29 | 0.61 |
| Tanzania | 1.77 | 11.26 | 0.00 | -0.86 | | 6.38 | -0.57 | 0.64 |
| Thailand | 1.90 | 11.99 | 0.02 | -0.77 | | 8.88 | -0.25 | 0.63 |
| Togo | 1.77 | 9.01 | 0.08 | -0.88 | | 7.30 | -0.83 | 0.51 |
| Trinidad and Tobago | 1.78 | 11.68 | 0.17 | -0.55 | | 9.23 | 0.12 | 0.73 |
| Tunisia | 4.56 | 18.23 | 0.05 | 0.09 | | 8.85 | -0.18 | 0.57 |
| Turkey | 5.55 | 22.57 | 0.21 | 0.80 | | 8.86 | -0.47 | 0.51 |
| Turkmenistan | 5.55 | 20.91 | 0.22 | 0.70 | | 8.36 | -1.44 | 0.65 |
| Uganda | 1.75 | 9.87 | 0.26 | -0.52 | | 7.21 | -0.61 | 0.69 |
| Ukraine | 7.96 | 21.29 | 0.33 | 1.25 | | 8.52 | -0.70 | 0.51 |
| United Arab Emirates | 2.91 | 16.05 | 0.00 | -0.37 | | 9.97 | 0.78 | 0.75 |
| United Kingdom | 10.83 | 15.77 | 0.58 | 1.68 | | 10.20 | 1.51 | 0.74 |
| United States | 5.17 | 24.53 | 0.59 | 1.53 | | 10.51 | 1.40 | 0.76 |
| Uruguay | 4.33 | 17.84 | 0.67 | 1.07 | | 9.08 | 0.96 | 0.67 |
| Uzbekistan | 6.07 | 20.91 | 0.22 | 0.77 | | 7.60 | -1.01 | 0.73 |
| Venezuela | 1.77 | 10.79 | 0.09 | -0.74 | | 8.66 | -1.89 | 0.66 |
| Vietnam | 1.81 | 13.73 | 0.05 | -0.60 | | 7.76 | -0.35 | 0.59 |
| Yemen | 1.86 | 8.26 | 0.00 | -1.06 | | 6.78 | -2.13 | 0.56 |
| Zambia | 1.80 | 13.00 | 0.00 | -0.74 | | 6.75 | -0.38 | 0.71 |
| Zimbabwe | 2.31 | 13.76 | 0.00 | -0.61 | | 7.77 | -1.28 | 0.63 |
|  |  |  |  |  | |  |  |  |
| ^a^Day-length variability is the absolute difference between day-length hours on 21 June and 21 December, computed by the Chronology Unit of the University of Groningen.  ^b^Temperature variability is the standard deviation of the lowest and highest temperatures in the coldest and hottest months [retrieved from Parker, P. M. *National cultures of the world: a statistical reference* (Greenwood Press, 1997)].  ^c^Daily-precipitation variability, which is greater to the extent that it is neither predominantly dry nor predominantly wet, is proxied by the minimal number of rain days per month divided by the maximal number of rain days per month [retrieved from Parker, P. M. *National cultures of the world: a statistical reference* (Greenwood Press, 1997)].  ^d^Common factor of the standardized variabilities in day length, temperature, and daily precipitation (eigenvalue λ = 2.189, *R^2^* = 0.729; Cronbach’s α = 0.813).  ^e^National wealth is the natural log of the average income per head in 2000, 2002, and 2004 [retrieved from United Nations, *Human Development Report* (Oxford Univ. Press, 2002, 2004, 2006)].  ^f^The World Bank’s index of governance quality (<https://info.worldbank.org/governance/wgi> 1996-2018), consisting of the following components: regulatory quality, government effectiveness, rule of law, control of corruption, voice and accountability, and political stability (eigenvalue λ = 5.208, *R^2^* = 0.868; Cronbach’s α = 0.969).  ^g^Index of psychosocial or eudaimonic well-being, assessed through annual Gallup World Polls from 2010 to 2015 [source: Joshanloo, M. Optimal human functioning around the world: a new index of eudaimonic well-being in 166 nations. *Br. J. Psychol.* **109**, 637-655 (2018)]. As elaborated in Supplementary Methods M4, this index consists of three psychic components (freedom to choose what one does with one’s life, and opportunities for learning and getting ahead), and four social components (helping people in need, volunteering, and receiving social support and respectful treatment) (eigenvalue λ = 3.037, *R^2^* = 0.434; Cronbach’s α = 0.774). | | | | | | | | |

**S5 Table | Explanatory ecological model: main and interaction effects of habitat variability on perceived livability for minorities (total *N* = 163), broken down by hemisphere.**

|  |  | |  | |  | | | |  |  | |
| --- | --- | --- | --- | --- | --- | --- | --- | --- | --- | --- | --- |
| Global area | Hemisphere | | | | | | | | | Entire world | |
|  |  | |  | |  | | | |  |  |  |
|  | North | | South | | East | | | | West |  |  |
|  |  | |  | | |  | |  | |  | |
| Number of countries | 132 | | 31 | | | 122 | | 41 | | 163 | |
|  |  | |  | | |  | |  | |  | |
| Habitat variability (HV) | | 0.019  (-0.003, 0.042) | | 0.072  (-0.007, 0.151) | | | 0.036^**^  (0.013, 0.060) | 0.048  (-0.013, 0.108) | | 0.023^*^  (0.002, 0.044) |  |
| National wealth (ln) (NW) | | 0.070^***^  (0.048, 0.092) | | 0.111^**^  (0.043, 0.179) | | | 0.062^***^  (0.040, 0.085) | 0.088^***^  (0.039, 0.136) | | 0.078^***^  (0.058, 0.098) |  |
| HV * NW | | 0.033^***^  (0.014, 0.053) | | 0.047  (-0.019, 0.113) | | | 0.031^**^  (0.011, 0.052) | -0.004  (-0.042, 0.035) | | 0.028^**^  (0.010, 0.046) |  |
|  | |  | |  | | |  |  | |  |  |
| *R^2^* (Δ*R^2^* HV * NW) | | 0.477^***^ (0.048) | | 0.695^***^ (0.024) | | | 0.486^***^ (0.040) | 0.747^***^ (0.000) | | 0.499^***^ (0.031) |  |
|  |  | |  | | |  | |  | |  | |
| Shown are unstandardized regression coefficients (*B*) with 95% confidence intervals between brackets. ^*^*P* < 0.05. ^**^*P* < 0.01. ^***^*P* < 0.001. | | | | | | | | | | | |

**S6 Table | Explanatory ecological model: main and interaction effects of habitat variability on perceived livability for minorities (*N* = 163), broken down by component of variability.**

|  | |  | |  | | |  | |  | | |  |
| --- | --- | --- | --- | --- | --- | --- | --- | --- | --- | --- | --- | --- |
| Predictor | | Component of variability | | | | | | | | | |  |
|  |  |  | |  | | |  | |  | | |  |
|  |  | Day length | | Temperature | | | Daily precipitation | | Total | | |  |
|  | |  | | |  | |  | | |  | |  |
| Habitat variability (HV) | | 0.012  (-0.013, 0.037) | | | 0.007  (-0.012, 0.025) | | 0.025^*^  (0.001, 0.050) | | | 0.023^*^  (0.002, 0.044) | |  |
| National wealth (ln) (NW) | | 0.083^***^  (0.063, 0.104) | | | 0.091^***^  (0.073, 0.110) | | 0.075^***^  (0.055, 0.095) | | | 0.078^***^  (0.058, 0.098) | |  |
| HV * NW | | 0.025^**^  (0.006, 0.045) | | | 0.008  (-0.011, 0.027) | | 0.029^**^  (0.010, 0.049) | | | 0.028^**^  (0.010, 0.046) | |  |
|  | |  | | |  | |  | | |  | |  |
| *R^2^* (Δ*R^2^* HV * NW) | | 0.487^***^ (0.021) | | | 0.433^***^ (0.003) | | 0.531^***^ (0.025) | | | 0.499^***^ (0.031) | |  |
|  |  | |  | | |  | |  | | |  | |
| Shown are unstandardized regression coefficients (*B*) with 95% confidence intervals between brackets. ^*^*P* < 0.05. ^**^*P* < 0.01. ^***^*P* < 0.001. | | | | | | | | | | | |  |

**S7 Table | Explanatory ecological model: main and interaction effects of habitat variability on perceived livability for minorities (*N* = 163), broken down by minority group.**

|  |  |  | |  |  |  |
| --- | --- | --- | --- | --- | --- | --- |
| Predictor | Perceived livability for minorities | | | | | |
|  |  |  | |  |  |  |
|  | Racial and ethnic minorities | | Gay or lesbian people | Immigrants from other countries | Intellectually disabled people | Overall  livability |
|  |  |  | |  |  |  |
| Habitat variability (HV) | 0.010  (-0.015, 0.035) | | 0.034^*^  (0.002, 0.067) | 0.007  (-0.016, 0.031) | 0.039^***^  (0.015, 0.063) | 0.023^*^  (0.002, 0.044) |
| National wealth (ln) (NW) | 0.029^*^  (0.005, 0.052) | | 0.171^***^  (0.141, 0.201) | 0.033^**^  (0.011, 0.056) | 0.079^***^  (0.056, 0.101) | 0.078^***^  (0.058, 0.098) |
| HV * NW | 0.027^*^  (0.006, 0.049) | | 0.036^**^  (0.009, 0.063) | 0.034^***^  (0.014, 0.054) | 0.016  (-0.005, 0.036) | 0.028^**^  (0.010, 0.046) |
|  |  | |  |  |  |  |
| *R^2^* (Δ*R^2^* HV * NW) | 0.137^***^ (0.036) | | 0.632^***^ (0.016) | 0.181^***^ (0.058) | 0.484^***^ (0.008) | 0.499^***^ (0.031) |
|  |  |  | |  |  |  |
| Shown are unstandardized regression coefficients (*B*) with 95% confidence intervals between brackets. ^*^*P* < 0.05. ^**^*P* < 0.01. ^***^*P* < 0.001. | | | | | | |

**S8 Table | Explanatory ecological model: main and interaction effects of habitat variability on perceived livability for racial and ethnic minorities mediated by governance quality rather than own habitat satisfaction (*R^2^* = 0.243; *N* = 163).**

|  |  |  |  |  |  |
| --- | --- | --- | --- | --- | --- |
| Predictor | *B* | s.e. | *P* | LLCI | ULCI |
|  |  |  |  |  |  |
| Habitat variability (HV) | 0.003 | 0.009 | 0.749 | -0.014 | 0.020 |
| Governance quality | 0.039 | 0.010 | < 0.001 | 0.018 | 0.059 |
| Own habitat satisfaction | 0.003 | 0.001 | 0.007 | 0.001 | 0.004 |
|  | | | | | |
| Conditional indirect effect of habitat variability via governance quality  (index of modified mediation = 0.008, CI = 0.004 to 0.013) | | | | | |
| Predictor | *B* | Boot s.e. |  | LLCI | ULCI |
|  |  |  |  |  |  |
| Poor (NW -1 s.d.) | -0.007 | 0.004 |  | -0.016 | 0.000 |
| Intermediate (mean NW) | 0.002 | 0.003 |  | -0.002 | 0.008 |
| Rich (NW +1 s.d.) | 0.011 | 0.004 |  | 0.005 | 0.020 |
|  |  |  |  |  |  |
| Conditional indirect effect of habitat variability via own habitat satisfaction  (index of modified mediation = 0.002, CI = -0.002 to 0.006) | | | | | |
| Predictor | *B* | Boot s.e. |  | LLCI | ULCI |
|  |  |  |  |  |  |
| Poor (NW -1 s.d.) | -0.002 | 0.005 |  | -0.012 | 0.008 |
| Intermediate (mean NW) | 0.000 | 0.003 |  | -0.006 | 0.007 |
| Rich (NW +1 s.d.) | 0.002 | 0.003 |  | -0.002 | 0.008 |

**S9 Table | Explanatory ecological model: main and interaction effects of habitat variability on perceived livability for gays and lesbians mediated by governance quality rather than own habitat satisfaction (*R^2^* = 0.664; *N* = 163).**

|  |  |  |  |  |  |
| --- | --- | --- | --- | --- | --- |
| Predictor | *B* | s.e. | *P* | LLCI | ULCI |
|  |  |  |  |  |  |
| Habitat variability (HV) | 0.060 | 0.014 | < 0.001 | 0.032 | 0.088 |
| Governance quality | 0.089 | 0.015 | < 0.001 | 0.060 | 0.118 |
| Own habitat satisfaction | 0.008 | 0.001 | < 0.001 | 0.006 | 0.010 |
|  | | | | | |
| Conditional indirect effect of habitat variability via governance quality  (index of modified mediation = 0.018, CI = 0.011 to 0.026) | | | | | |
| Predictor | *B* | Boot s.e. |  | LLCI | ULCI |
|  |  |  |  |  |  |
| Poor (NW -1 s.d.) | -0.015 | 0.008 |  | -0.033 | 0.000 |
| Intermediate (mean NW) | 0.005 | 0.006 |  | -0.005 | 0.017 |
| Rich (NW +1 s.d.) | 0.026 | 0.006 |  | 0.015 | 0.030 |
|  |  |  |  |  |  |
| Conditional indirect effect of habitat variability via own habitat satisfaction  (index of modified mediation = 0.005, CI = -0.007 to 0.018) | | | | | |
| Predictor | *B* | Boot s.e. |  | LLCI | ULCI |
|  |  |  |  |  |  |
| Poor (NW -1 s.d.) | -0.005 | 0.015 |  | -0.036 | 0.024 |
| Intermediate (mean NW) | 0.001 | 0.009 |  | -0.018 | 0.019 |
| Rich (NW +1 s.d.) | 0.007 | 0.007 |  | -0.008 | 0.022 |

**S10 Table | Explanatory ecological model: main and interaction effects of habitat variability on perceived livability for foreign immigrants mediated by governance quality rather than own habitat satisfaction (*R^2^* = 0.177; *N* = 163).**

|  |  |  |  |  |  |
| --- | --- | --- | --- | --- | --- |
| Predictor | *B* | s.e. | *P* | LLCI | ULCI |
|  |  |  |  |  |  |
| Habitat variability (HV) | 0.012 | 0.010 | 0.244 | -0.008 | 0.033 |
| Governance quality | 0.043 | 0.012 | < 0.001 | 0.020 | 0.066 |
| Own habitat satisfaction | 0.000 | 0.001 | 0.997 | -0.002 | 0.002 |
|  | | | | | |
| Conditional indirect effect of habitat variability via governance quality  (index of modified mediation = 0.009, CI = 0.004 to 0.015) | | | | | |
| Predictor | *B* | Boot s.e. |  | LLCI | ULCI |
|  |  |  |  |  |  |
| Poor (NW -1 s.d.) | -0.007 | 0.004 |  | -0.017 | 0.000 |
| Intermediate (mean NW) | 0.003 | 0.003 |  | -0.003 | 0.009 |
| Rich (NW +1 s.d.) | 0.013 | 0.004 |  | 0.006 | 0.022 |
|  |  |  |  |  |  |
| Conditional indirect effect of habitat variability via own habitat satisfaction  (index of modified mediation = 0.000, CI = -0.002 to 0.002) | | | | | |
| Predictor | *B* | Boot s.e. |  | LLCI | ULCI |
|  |  |  |  |  |  |
| Poor (NW -1 s.d.) | 0.000 | 0.002 |  | -0.004 | 0.003 |
| Intermediate (mean NW) | 0.000 | 0.001 |  | -0.002 | 0.002 |
| Rich (NW +1 s.d.) | 0.000 | 0.001 |  | -0.002 | 0.003 |

**S11 Table | Explanatory ecological model: main and interaction effects of habitat variability on perceived livability for people with intellectual disabilities mediated by governance quality rather than own habitat satisfaction (*R^2^* = 0.545; *N* = 163).**

|  |  |  |  |  |  |
| --- | --- | --- | --- | --- | --- |
| Predictor | *B* | s.e. | *P* | LLCI | ULCI |
|  |  |  |  |  |  |
| Habitat variability (HV) | 0.041 | 0.010 | < 0.001 | 0.022 | 0.061 |
| Governance quality | 0.060 | 0.011 | < 0.001 | 0.038 | 0.082 |
| Own habitat satisfaction | 0.003 | 0.001 | < 0.001 | 0.001 | 0.005 |
|  | | | | | |
| Conditional indirect effect of habitat variability via governance quality  (index of modified mediation = 0.012, CI = 0.007 to 0.016) | | | | | |
| Predictor | *B* | Boot s.e. |  | LLCI | ULCI |
|  |  |  |  |  |  |
| Poor (NW -1 s.d.) | -0.010 | 0.006 |  | -0.023 | 0.000 |
| Intermediate (mean NW) | 0.000 | 0.004 |  | -0.003 | 0.012 |
| Rich (NW +1 s.d.) | 0.018 | 0.005 |  | 0.010 | 0.028 |
|  |  |  |  |  |  |
| Conditional indirect effect of habitat variability via own habitat satisfaction  (index of modified mediation = 0.002, CI = -0.003 to 0.007) | | | | | |
| Predictor | *B* | Boot s.e. |  | LLCI | ULCI |
|  |  |  |  |  |  |
| Poor (NW -1 s.d.) | -0.002 | 0.006 |  | -0.014 | 0.010 |
| Intermediate (mean NW) | 0.000 | 0.004 |  | -0.006 | 0.008 |
| Rich (NW +1 s.d.) | 0.003 | 0.003 |  | -0.003 | 0.010 |

**S12 Table | Explanatory ecological model: main and interaction effects of habitat variability on perceived livability for minorities controlled for ecological covariates (*R^2^* = 0.533; *N* = 150)^a^.**

|  |  | |  | |  | |  | |  | |
| --- | --- | --- | --- | --- | --- | --- | --- | --- | --- | --- |
| Predictor | *B* | | s.e. | | *P* | | LLCI | | ULCI | |
|  |  | |  | |  | |  | |  | |
| Habitat variability (HV) | 0.028 | | 0.014 | | 0.039 | | 0.001 | | 0.055 | |
| National wealth (ln) (NW) | 0.064 | | 0.018 | | < 0.001 | | 0.029 | | 0.099 | |
| HV * NW | 0.026 | | 0.010 | | 0.007 | | 0.007 | | 0.045 | |
| Natural disasters (ln) | 0.001 | | 0.003 | | 0.708 | | -0.005 | | 0.008 | |
| Pathogenic diseases | -0.000 | | 0.007 | | 0.991 | | -0.014 | | 0.014 | |
| Agricultural subsistence | -0.000 | | 0.001 | | 0.729 | | -0.001 | | 0.001 | |
| Urbanization | -0.000 | | 0.001 | | 0.996 | | -0.001 | | 0.001 | |
|  |  |  | |  | |  | |  | |  |
|  |  |  | |  | |  | |  | |  |
| ^a^A highly similar pattern of results was obtained for each of the targeted minority groups. | | | | | | | | | | |

**S13 Table | Explanatory ecological model: main and interaction effects of habitat variability on perceived livability for minorities controlled for demographic covariates (*R^2^* = 0.531; *N* = 140)^a^.**

|  |  | |  | |  | |  | |  | |
| --- | --- | --- | --- | --- | --- | --- | --- | --- | --- | --- |
| Predictor | *B* | | s.e. | | *P* | | LLCI | | ULCI | |
|  |  | |  | |  | |  | |  | |
| Habitat variability (HV) | 0.034 | | 0.014 | | 0.032 | | 0.013 | | 0.007 | |
| National wealth (ln) (NW) | 0.068 | | 0.013 | | < 0.001 | | 0.042 | | 0.094 | |
| HV * NW | 0.024 | | 0.010 | | 0.015 | | 0.005 | | 0.043 | |
| Population density (ln) | 0.000 | | 0.000 | | 0.486 | | -0.000 | | 0.000 | |
| Ethnic diversity | -0.013 | | 0.043 | | 0.770 | | -0.097 | | 0.072 | |
| Religious diversity | 0.024 | | 0.039 | | 0.529 | | -0.052 | | 0.101 | |
| Immigration rate (truncated) | 0.000 | | 0.001 | | 0.787 | | -0.002 | | 0.003 | |
| Income inequality | 0.001 | | 0.001 | | 0.536 | | -0.002 | | 0.003 | |
|  |  |  | |  | |  | |  | |  |
| ^a^A highly similar pattern of results was obtained for each of the targeted minority groups. | | | | | | | | | | |

**S14 Table | Prospective model: predictors, predictions, and the criterion measure of perceived livability for minorities.**

|  |  | |  | | |  | | |  | |  | | |  | | |  | |  |
| --- | --- | --- | --- | --- | --- | --- | --- | --- | --- | --- | --- | --- | --- | --- | --- | --- | --- | --- | --- |
|  |  | | |  | | | |  | | | |  | | | |  | | |  |
| Country | Latitude^a^ | | Longitude | | | Habitat variability^b^ | | | National wealth^c^ | | Future degree of livability for minorities | | | | | | | |  |
|  |  |  |  |  |  |  |  |  |  |  | Prediction 1^d^ | | | Prediction 2^e^ | | | Measure^f^ | |  |
|  |  | |  | | |  | | |  | |  | | |  | | |  | |  |
| Afghanistan | 33.94 | | 67.71 | | | 0.25 | | | 6.81 | | -0.03 | | | 0.12 | | | 0.55 | |  |
| Albania | 41.15 | | 20.17 | | | 0.40 | | | 8.40 | | -0.10 | | | 0.00 | | | 0.47 | |  |
| Algeria | 28.03 | | 1.66 | | | -0.18 | | | 8.68 | | -0.03 | | | 0.00 | | | 0.53 | |  |
| Angola | -11.20 | | 17.87 | | | -1.05 | | | 7.68 | | -0.01 | | | 0.06 | | |  | |  |
| Argentina | -38.42 | | -63.62 | | | 0.95 | | | 9.41 | | -0.21 | | | -0.09 | | | 0.21 | |  |
| Armenia | 40.07 | | 45.04 | | | 0.82 | | | 8.09 | | -0.08 | | | 0.02 | | | 0.45 | |  |
| Australia | -25.27 | | 133.78 | | | 1.16 | | | 10.24 | | -0.03 | | | -0.18 | | | 0.09 | |  |
| Austria | 47.52 | | 14.55 | | | 1.47 | | | 10.29 | | -0.14 | | | -0.20 | | | 0.22 | |  |
| Azerbaijan | 40.14 | | 47.58 | | | 0.82 | | | 8.14 | | -0.08 | | | 0.02 | | | 0.36 | |  |
| Bahrain | 25.93 | | 50.64 | | | -0.44 | | | 9.78 | | 0.00 | | | -0.05 | | | 0.38 | |  |
| Bangladesh | 23.68 | | 90.36 | | | -0.60 | | | 7.45 | | 0.02 | | | 0.07 | | | 0.22 | |  |
| Belarus | 53.71 | | 27.95 | | | 1.76 | | | 8.81 | | -0.19 | | | -0.07 | | | 0.44 | |  |
| Belgium | 50.50 | | 4.47 | | | 1.59 | | | 10.26 | | -0.17 | | | -0.21 | | | 0.22 | |  |
| Belize | 17.19 | | -88.50 | | | -0.54 | | | 8.72 | | -0.04 | | | 0.00 | | |  | |  |
| Benin | 9.31 | | 2.32 | | | -1.11 | | | 6.96 | | 0.01 | | | 0.09 | | | 0.39 | |  |
| Bhutan | 27.51 | | 90.43 | | | -0.43 | | | 7.43 | | 0.01 | | | 0.07 | | |  | |  |
| Bolivia | -16.29 | | -63.59 | | | -0.63 | | | 7.84 | | -0.06 | | | 0.05 | | | 0.33 | |  |
| Bosnia | 43.92 | | 17.68 | | | 1.27 | | | 8.78 | | -0.12 | | | -0.05 | | | 0.51 | |  |
| Botswana | -22.33 | | 24.68 | | | -0.26 | | | 9.04 | | -0.06 | | | -0.02 | | | 0.35 | |  |
| Brazil | -14.24 | | -51.93 | | | -0.34 | | | 8.97 | | -0.05 | | | -0.01 | | | 0.18 | |  |
| Bulgaria | 42.73 | | 25.49 | | | 0.98 | | | 8.85 | | -0.10 | | | -0.05 | | | 0.39 | |  |
| Burkina Faso | 12.24 | | -1.56 | | | -0.57 | | | 6.99 | | 0.01 | | | 0.09 | | | 0.38 | |  |
| Burundi | -3.37 | | 29.92 | | | -1.19 | | | 6.45 | | 0.02 | | | 0.10 | | | 0.45 | |  |
| Cambodia | 12.57 | | 104.99 | | | -0.87 | | | 7.59 | | 0.05 | | | 0.06 | | | 0.47 | |  |
| Cameroon | 7.37 | | 12.35 | | | -0.87 | | | 7.58 | | 0.02 | | | 0.06 | | | 0.43 | |  |
| Canada | 56.13 | | -106.35 | | | 2.63 | | | 10.29 | | -0.27 | | | -0.28 | | | 0.08 | |  |
| Central African Republic | 6.61 | | 20.94 | | | -0.90 | | | 7.04 | | 0.02 | | | 0.09 | | | 0.56 | |  |
| Chad | 15.45 | | 18.73 | | | -0.61 | | | 7.19 | | 0.01 | | | 0.08 | | | 0.45 | |  |
| Chile | -35.68 | | -71.54 | | | -0.02 | | | 9.21 | | -0.19 | | | -0.04 | | | 0.27 | |  |
| China | 35.86 | | 104.20 | | | 0.44 | | | 8.48 | | -0.03 | | | 0.00 | | | 0.35 | |  |
| Colombia | 4.57 | | -74.30 | | | -0.50 | | | 8.80 | | -0.02 | | | 0.00 | | | 0.33 | |  |
| Comoros | -11.88 | | 43.87 | | | -0.95 | | | 7.46 | | 0.00 | | | 0.07 | | | 0.38 | |  |
| Congo | -0.23 | | 15.83 | | | -0.95 | | | 6.83 | | 0.01 | | | 0.09 | | | 0.46 | |  |
| Costa Rica | 9.75 | | -83.75 | | | -0.92 | | | 9.10 | | -0.02 | | | 0.00 | | | 0.26 | |  |
| Croatia | 45.10 | | 15.20 | | | 1.57 | | | 9.23 | | -0.13 | | | -0.10 | | | 0.45 | |  |
| Cyprus | 35.13 | | 33.43 | | | 0.04 | | | 9.93 | | -0.05 | | | -0.09 | | | 0.30 | |  |
| Czech Republic | 49.82 | | 15.47 | | | 1.37 | | | 9.78 | | -0.16 | | | -0.15 | | | 0.34 | |  |
| Democratic Congo | -4.04 | | 21.76 | | | -1.00 | | | 6.56 | | 0.01 | | | 0.11 | | | 0.50 | |  |
| Denmark | 56.26 | | 9.50 | | | 1.85 | | | 10.31 | | -0.22 | | | -0.23 | | | 0.12 | |  |
| Djibouti | 11.83 | | 42.59 | | | -0.85 | | | 7.60 | | 0.03 | | | 0.06 | | |  | |  |
| Dominican Republic | 18.74 | | -70.16 | | | -0.62 | | | 8.81 | | -0.03 | | | 0.00 | | | 0.39 | |  |
| Ecuador | -1.83 | | -78.18 | | | -0.54 | | | 8.18 | | -0.03 | | | 0.03 | | | 0.32 | |  |
| Egypt | 26.82 | | 30.80 | | | -0.23 | | | 8.26 | | -0.01 | | | 0.02 | | | 0.47 | |  |
| El Salvador | 13.79 | | -88.90 | | | -0.63 | | | 8.48 | | -0.03 | | | 0.02 | | | 0.34 | |  |
| Estonia | 58.60 | | 25.01 | | | 2.38 | | | 9.42 | | -0.24 | | | -0.16 | | | 0.40 | |  |
| Eswatini | -26.52 | | 31.47 | | | -0.16 | | | 8.50 | | -0.08 | | | 0.01 | | | 0.42 | |  |
| Ethiopia | 9.15 | | 40.49 | | | -0.65 | | | 6.60 | | 0.03 | | | 0.11 | | | 0.43 | |  |
| Finland | 61.92 | | 25.75 | | | 2.88 | | | 10.21 | | -0.27 | | | -0.28 | | | 0.17 | |  |
| France | 46.23 | | 2.21 | | | 1.41 | | | 10.20 | | -0.14 | | | -0.19 | | | 0.19 | |  |
| Gabon | -0.80 | | 11.61 | | | -1.03 | | | 8.78 | | 0.01 | | | 0.02 | | | 0.46 | |  |
| Georgia | 42.32 | | 43.36 | | | 0.69 | | | 7.86 | | -0.09 | | | 0.04 | | | 0.37 | |  |
| Germany | 51.17 | | 10.45 | | | 1.73 | | | 10.20 | | -0.18 | | | -0.21 | | | 0.15 | |  |
| Ghana | 7.95 | | -1.02 | | | -0.88 | | | 7.66 | | 0.01 | | | 0.06 | | | 0.47 | |  |
| Greece | 39.07 | | 21.82 | | | 0.24 | | | 9.86 | | -0.08 | | | -0.09 | | | 0.51 | |  |
| Guatemala | 15.78 | | -90.23 | | | -0.84 | | | 8.31 | | -0.03 | | | 0.03 | | | 0.42 | |  |
| Guinea | 9.95 | | -9.70 | | | -1.11 | | | 7.64 | | 0.01 | | | 0.06 | | | 0.43 | |  |
| Haiti | 18.97 | | -72.29 | | | -0.69 | | | 7.41 | | -0.03 | | | 0.07 | | | 0.54 | |  |
| Honduras | 15.20 | | -86.24 | | | -1.20 | | | 7.88 | | -0.03 | | | 0.05 | | | 0.38 | |  |
| Hong Kong | 22.40 | | 114.11 | | | -0.46 | | | 10.23 | | 0.04 | | | -0.08 | | | 0.29 | |  |
| Hungary | 47.16 | | 19.50 | | | 1.47 | | | 9.56 | | -0.14 | | | -0.13 | | | 0.46 | |  |
| Iceland | 64.96 | | -19.02 | | | 2.29 | | | 10.34 | | -0.33 | | | -0.26 | | | 0.06 | |  |
| India | 20.59 | | 78.96 | | | -0.34 | | | 7.91 | | 0.03 | | | 0.05 | | | 0.32 | |  |
| Indonesia | -0.79 | | 113.92 | | | -0.87 | | | 8.10 | | 0.06 | | | 0.04 | | | 0.52 | |  |
| Iran | 32.43 | | 53.69 | | | 0.40 | | | 8.81 | | -0.03 | | | -0.03 | | | 0.45 | |  |
| Iraq | 33.22 | | 43.68 | | | 0.14 | | | 8.43 | | -0.04 | | | 0.01 | | | 0.48 | |  |
| Ireland | 53.41 | | -8.24 | | | 1.57 | | | 10.46 | | -0.20 | | | -0.23 | | | 0.08 | |  |
| Israel | 31.05 | | 34.85 | | | -0.16 | | | 9.97 | | -0.03 | | | -0.08 | | | 0.48 | |  |
| Italy | 41.87 | | 12.57 | | | 0.31 | | | 10.17 | | -0.10 | | | -0.12 | | | 0.26 | |  |
| Ivory Coast | 7.54 | | -5.55 | | | -0.92 | | | 7.36 | | 0.01 | | | 0.07 | | | 0.39 | |  |
| Jamaica | 18.11 | | -77.30 | | | -0.82 | | | 8.28 | | -0.03 | | | 0.03 | | | 0.27 | |  |
| Japan | 36.20 | | 138.25 | | | 0.30 | | | 10.23 | | -0.01 | | | -0.12 | | | 0.29 | |  |
| Jordan | 30.59 | | 36.24 | | | -0.10 | | | 8.36 | | -0.03 | | | 0.02 | | | 0.45 | |  |
| Kazakhstan | 48.02 | | 66.92 | | | 1.61 | | | 8.76 | | -0.13 | | | -0.06 | | | 0.34 | |  |
| Kenya | -0.02 | | 37.91 | | | -0.79 | | | 6.97 | | 0.02 | | | 0.09 | | | 0.40 | |  |
| Kosovo | 42.60 | | 20.90 | | | 1.23 | | | 8.61 | | -0.10 | | | -0.03 | | | 0.42 | |  |
| Kuwait | 29.31 | | 47.48 | | | -0.21 | | | 9.75 | | -0.02 | | | -0.06 | | | 0.41 | |  |
| Kyrgyzstan | 41.20 | | 74.77 | | | 1.12 | | | 7.64 | | -0.07 | | | 0.06 | | | 0.39 | |  |
| Laos | 19.86 | | 102.50 | | | -0.59 | | | 7.47 | | 0.04 | | | 0.07 | | | 0.31 | |  |
| Latvia | 56.88 | | 24.60 | | | 2.20 | | | 9.14 | | -0.22 | | | -0.12 | | | 0.36 | |  |
| Lebanon | 33.85 | | 35.86 | | | -0.19 | | | 8.48 | | -0.04 | | | 0.01 | | | 0.47 | |  |
| Lesotho | -29.61 | | 28.23 | | | 0.02 | | | 7.77 | | -0.10 | | | 0.05 | | | 0.40 | |  |
| Liberia | 6.43 | | -9.43 | | | -1.03 | | | 6.45 | | 0.01 | | | 0.11 | | | 0.52 | |  |
| Libya | 26.34 | | 17.23 | | | -0.29 | | | 8.93 | | -0.02 | | | -0.01 | | | 0.32 | |  |
| Lithuania | 55.17 | | 23.88 | | | 2.06 | | | 9.23 | | -0.21 | | | -0.12 | | | 0.36 | |  |
| Luxembourg | 49.82 | | 6.13 | | | 1.53 | | | 11.01 | | -0.17 | | | -0.28 | | | 0.11 | |  |
| Madagascar | -18.77 | | 46.87 | | | -0.62 | | | 6.70 | | -0.03 | | | 0.11 | | | 0.50 | |  |
| Malawi | -13.25 | | 34.30 | | | -0.69 | | | 6.42 | | -0.01 | | | 0.12 | | | 0.60 | |  |
| Malaysia | 4.21 | | 101.98 | | | -0.50 | | | 9.16 | | 0.05 | | | -0.02 | | | 0.63 | |  |
| Mali | 17.57 | | -4.00 | | | -0.55 | | | 6.81 | | 0.00 | | | 0.10 | | | 0.38 | |  |
| Malta | 35.94 | | 14.38 | | | -0.34 | | | 9.81 | | -0.07 | | | -0.06 | | | 0.18 | |  |
| Mauritania | 21.01 | | -10.94 | | | -0.51 | | | 7.57 | | -0.01 | | | 0.06 | | | 0.54 | |  |
| Mauritius | -20.35 | | 57.55 | | | -0.89 | | | 9.30 | | -0.03 | | | -0.01 | | | 0.24 | |  |
| Mexico | 23.63 | | -102.55 | | | -0.50 | | | 9.13 | | -0.06 | | | -0.02 | | | 0.33 | |  |
| Moldova | 47.41 | | 28.37 | | | 1.21 | | | 7.48 | | -0.14 | | | 0.07 | | | 0.56 | |  |
| Mongolia | 46.86 | | 103.85 | | | 1.13 | | | 7.52 | | -0.10 | | | 0.07 | | | 0.49 | |  |
| Montenegro | 42.71 | | 19.37 | | | 1.49 | | | 8.92 | | -0.11 | | | -0.07 | | | 0.43 | |  |
| Morocco | 31.79 | | -7.09 | | | -0.06 | | | 8.27 | | -0.05 | | | 0.02 | | | 0.43 | |  |
| Mozambique | -18.67 | | 35.53 | | | -0.36 | | | 6.95 | | -0.03 | | | 0.10 | | | 0.28 | |  |
| Myanmar | 21.91 | | 95.96 | | | -0.76 | | | 6.93 | | 0.03 | | | 0.09 | | | 0.37 | |  |
| Nagorno Karabakh | 39.80 | | 46.66 | | | 0.78 | | | 8.14 | | -0.08 | | | 0.02 | | |  | |  |
| Namibia | -22.96 | | 18.49 | | | -0.43 | | | 8.81 | | -0.06 | | | 0.00 | | | 0.42 | |  |
| Nepal | 28.39 | | 84.12 | | | -0.41 | | | 7.24 | | 0.00 | | | 0.08 | | | 0.12 | |  |
| Netherlands | 52.13 | | 5.29 | | | 1.70 | | | 10.27 | | -0.19 | | | -0.21 | | | 0.14 | |  |
| New Zealand | -40.90 | | 174.89 | | | 0.82 | | | 9.99 | | -0.13 | | | -0.14 | | | 0.07 | |  |
| Nicaragua | 12.87 | | -85.21 | | | -1.24 | | | 7.95 | | -0.03 | | | 0.05 | | | 0.29 | |  |
| Niger | 17.61 | | 8.08 | | | -0.55 | | | 6.65 | | 0.00 | | | 0.11 | | | 0.41 | |  |
| Nigeria | 9.08 | | 8.68 | | | -0.88 | | | 6.88 | | 0.02 | | | 0.09 | | | 0.43 | |  |
| North Macedonia | 41.61 | | 21.75 | | | 1.43 | | | 8.71 | | -0.10 | | | -0.05 | | | 0.50 | |  |
| Northern Cyprus | 35.25 | | 33.66 | | | 0.04 | | | 9.93 | | -0.05 | | | -0.09 | | | 0.36 | |  |
| Norway | 60.47 | | 8.47 | | | 2.23 | | | 10.46 | | -0.27 | | | -0.27 | | | 0.08 | |  |
| Pakistan | 30.38 | | 69.35 | | | 0.03 | | | 7.62 | | -0.01 | | | 0.06 | | | 0.48 | |  |
| Palestine | 31.65 | | 34.77 | | | -0.16 | | | 8.97 | | -0.03 | | | -0.02 | | | 0.50 | |  |
| Panama | 8.54 | | -80.78 | | | -1.01 | | | 8.78 | | -0.02 | | | 0.02 | | | 0.35 | |  |
| Paraguay | -23.44 | | -58.44 | | | 0.19 | | | 8.44 | | -0.10 | | | 0.00 | | | 0.35 | |  |
| Peru | -9.19 | | -75.02 | | | -0.98 | | | 8.55 | | -0.04 | | | 0.02 | | | 0.38 | |  |
| Philippines | 12.88 | | 121.77 | | | -0.93 | | | 8.36 | | 0.06 | | | 0.03 | | | 0.36 | |  |
| Poland | 51.92 | | 19.15 | | | 1.52 | | | 9.29 | | -0.18 | | | -0.11 | | | 0.47 | |  |
| Portugal | 39.40 | | -8.22 | | | -0.03 | | | 9.82 | | -0.10 | | | -0.08 | | | 0.13 | |  |
| Puerto Rico | 18.22 | | -66.59 | | | -0.37 | | | 9.85 | | -0.03 | | | -0.06 | | |  | |  |
| Qatar | 25.35 | | 51.18 | | | -0.33 | | | 9.90 | | 0.00 | | | -0.07 | | |  | |  |
| Romania | 45.94 | | 24.97 | | | 1.30 | | | 8.88 | | -0.13 | | | -0.06 | | | 0.49 | |  |
| Russia | 61.52 | | 105.32 | | | 2.67 | | | 9.09 | | -0.24 | | | -0.13 | | | 0.33 | |  |
| Rwanda | -1.94 | | 29.87 | | | -1.14 | | | 7.06 | | 0.02 | | | 0.08 | | | 0.56 | |  |
| Saudi Arabia | 23.89 | | 45.08 | | | -0.16 | | | 9.44 | | 0.00 | | | -0.05 | | | 0.20 | |  |
| Senegal | 14.50 | | -14.45 | | | -0.80 | | | 7.38 | | 0.00 | | | 0.07 | | | 0.43 | |  |
| Serbia | 44.02 | | 21.01 | | | 1.53 | | | 8.14 | | -0.11 | | | 0.01 | | | 0.37 | |  |
| Sierra Leone | 8.46 | | -11.78 | | | -1.18 | | | 6.26 | | 0.01 | | | 0.11 | | | 0.42 | |  |
| Singapore | 1.35 | | 103.82 | | | -0.02 | | | 10.13 | | 0.05 | | | -0.10 | | | 0.18 | |  |
| Slovakia | 48.67 | | 19.70 | | | 1.38 | | | 9.47 | | -0.15 | | | -0.12 | | | 0.45 | |  |
| Slovenia | 46.15 | | 15.00 | | | 1.61 | | | 9.89 | | -0.13 | | | -0.17 | | | 0.42 | |  |
| Somalia | 5.15 | | 46.20 | | | -1.09 | | | 6.66 | | 0.03 | | | 0.10 | | | 0.23 | |  |
| Somaliland | 5.15 | | 46.20 | | | -1.09 | | | 7.84 | | 0.03 | | | 0.05 | | |  | |  |
| South Africa | -30.56 | | 22.94 | | | -0.04 | | | 9.23 | | -0.11 | | | -0.04 | | | 0.33 | |  |
| South Korea | 35.91 | | 127.77 | | | 0.40 | | | 9.81 | | -0.02 | | | -0.10 | | | 0.34 | |  |
| South Sudan | 6.88 | | 31.31 | | | -0.46 | | | 7.53 | | 0.03 | | | 0.07 | | | 0.52 | |  |
| Spain | 40.46 | | -3.75 | | | 0.50 | | | 10.00 | | -0.10 | | | -0.12 | | | 0.13 | |  |
| Sri Lanka | 7.87 | | 80.77 | | | -0.77 | | | 8.25 | | 0.05 | | | 0.03 | | | 0.35 | |  |
| Sudan | 12.86 | | 30.22 | | | -0.46 | | | 7.53 | | 0.02 | | | 0.07 | | |  | |  |
| Suriname | 3.92 | | -56.03 | | | -0.63 | | | 8.64 | | -0.01 | | | 0.01 | | |  | |  |
| Sweden | 60.13 | | 18.64 | | | 2.28 | | | 10.19 | | -0.26 | | | -0.24 | | | 0.10 | |  |
| Switzerland | 46.82 | | 8.23 | | | 1.46 | | | 10.33 | | -0.14 | | | -0.20 | | | 0.17 | |  |
| Syria | 34.80 | | 39.00 | | | 0.04 | | | 8.19 | | -0.05 | | | 0.02 | | |  | |  |
| Taiwan | 23.70 | | 120.96 | | | -0.13 | | | 10.11 | | 0.03 | | | -0.09 | | | 0.21 | |  |
| Tajikistan | 38.86 | | 71.28 | | | 0.69 | | | 7.01 | | -0.06 | | | 0.11 | | | 0.19 | |  |
| Tanzania | -6.37 | | 34.89 | | | -0.86 | | | 6.38 | | 0.01 | | | 0.12 | | | 0.48 | |  |
| Thailand | 15.87 | | 100.99 | | | -0.77 | | | 8.88 | | 0.05 | | | 0.00 | | | 0.47 | |  |
| Togo | 8.62 | | 0.82 | | | -0.88 | | | 7.30 | | 0.01 | | | 0.08 | | | 0.40 | |  |
| Trinidad and Tobago | 10.69 | | -61.22 | | | -0.55 | | | 9.23 | | -0.01 | | | -0.02 | | | 0.30 | |  |
| Tunisia | 33.89 | | 9.54 | | | 0.09 | | | 8.85 | | -0.06 | | | -0.02 | | | 0.38 | |  |
| Turkey | 38.96 | | 35.24 | | | 0.80 | | | 8.86 | | -0.07 | | | -0.04 | | | 0.52 | |  |
| Turkmenistan | 38.97 | | 59.56 | | | 0.70 | | | 8.36 | | -0.06 | | | 0.00 | | | 0.27 | |  |
| Uganda | 1.37 | | 32.29 | | | -0.52 | | | 7.21 | | 0.02 | | | 0.08 | | | 0.48 | |  |
| Ukraine | 48.38 | | 31.17 | | | 1.25 | | | 8.52 | | -0.14 | | | -0.02 | | | 0.33 | |  |
| United Arab Emirates | 23.42 | | 53.85 | | | -0.37 | | | 9.97 | | 0.01 | | | -0.07 | | | 0.15 | |  |
| United Kingdom | 55.38 | | -3.44 | | | 1.68 | | | 10.20 | | -0.22 | | | -0.21 | | | 0.13 | |  |
| United States | 37.09 | | -95.71 | | | 1.53 | | | 10.51 | | -0.12 | | | -0.23 | | | 0.16 | |  |
| Uruguay | -32.52 | | -55.77 | | | 1.07 | | | 9.08 | | -0.16 | | | -0.07 | | | 0.14 | |  |
| Uzbekistan | 41.38 | | 64.59 | | | 0.77 | | | 7.60 | | -0.08 | | | 0.06 | | | 0.08 | |  |
| Venezuela | 6.42 | | -66.59 | | | -0.74 | | | 8.66 | | -0.02 | | | 0.01 | | | 0.34 | |  |
| Vietnam | 14.06 | | 108.28 | | | -0.60 | | | 7.76 | | 0.05 | | | 0.05 | | | 0.23 | |  |
| Yemen | 15.55 | | 48.52 | | | -1.06 | | | 6.78 | | 0.02 | | | 0.10 | | | 0.54 | |  |
| Zambia | -13.13 | | 27.85 | | | -0.74 | | | 6.75 | | -0.01 | | | 0.10 | | | 0.46 | |  |
| Zimbabwe | -19.02 | | 29.15 | | | -0.61 | | | 7.77 | | -0.04 | | | 0.05 | | | 0.49 | |  |
|  |  |  | | |  | |  | | |  | | |  | |  | | |  | |
| ^a^Midrange latitude and midrange longitude retrieved from [https://developers.google.­com/public-data/docs/canonical/countries_csv](https://developers.google.com/public-data/docs/canonical/countries_csv). ^b^Common factor of the variabilities in day length, temperature, and daily precipitation (eigenvalue λ = 2.19, *R^2^* = 0.73; Cronbach’s α = 0.81). ^c^National wealth is the natural log of the average income per head in 2000, 2002, and 2004 [retrieved from United Nations, *Human Development Report* (Oxford Univ. Press, 2002, 2004, 2006)].  ^d^Prediction based on the 2010-2015 regression equation for the descriptive geographical model: *Y* = (0.070 * *z*-latitude) + ((0.059) * (*z*-latitude * *z*-latitude)) + (-0.023 * *z*-longitude).  ^e^Prediction based on the 2010-2015 regression equation for the explanatory ecological model: *Y* = (0.023 * *z*-habitat variability) + (0.078 * *z*-national wealth) + ((0.028) * (*z*-habitat variability * *z*-national wealth)).  ^f^Country-level proportions of *not a good place to live* for racial and ethnic minorities, gay or lesbian people, and immigrants from other countries, retrieved from <https://www.gallup.com/analytics/318923/world-poll-public-datasets.aspx>. | | | | | | | | | | | | | | | | | | | |

**S15 Table | Explanatory ecological model: descriptive statistics of mediating and control variables, with sources in footnotes.**

|  |  |  |  |  |  |
| --- | --- | --- | --- | --- | --- |
| Society-level variable | *N* | Scale range | *M* (s.d.) | Skewness (s.e.) | Kurtosis (s.e.) |
|  |  |  |  |  |  |
| Governance quality^a^ | 160 | -2.28 to 2.01 | -0.069 (1.029) | 0.272 (0.192) | -0.487 (0.381) |
| Own habitat satisfaction ^b^ | 163 | 42.89 to 95.22 | 76.578 (12.465) | -0.582 (0.190) | -0.442 (0.378) |
| Natural disasters (ln)^c^ | 153 | 0.00 to 11.96 | 7.307 (3.198) | -0.740 (0.196) | -0.230 (0.390) |
| Pathogenic diseases^d^ | 160 | -2.18 to 4.73 | 0.430 (1.999) | 0.479 (0.192) | -1.101 (0.381) |
| Agricultural subsistence^e^ | 160 | 0.50 to 93.00 | 35.584 (28.298) | 0.537 (0.192) | -1.062 (0.381) |
| Urbanization^f^ | 158 | 13.70 to 100.00 | 61.266 (22.214) | -0.220 (0.193) | -0.883 (0.384) |
| Population density (ln)^g^ | 161 | 0.00 to 8.64 | 3.931 (1.468) | -0.011 (0.191) | 0.635 (0.380) |
| Ethnic diversity^h^ | 160 | 0.00 to 0.93 | 0.449 (0.256) | -0.040 (0.192) | -1.248 (0.381) |
| Religious diversity^h^ | 161 | 0.00 to 0.86 | 0.433 (0.234) | -0.144 (0.191) | -1.106 (0.380) |
| Immigration rate (capped)^i^ | 159 | -15.00 to 15.00 | -0.229 (5.379) | 0.282 (0.192) | 1.992 (0.383) |
| Income inequality^j^ | 140 | 16.8 to 74.3 | 40.326 (9.531) | 0.584 (0.205) | 0.469 (0.407) |
|  |  |  |  |  |  |
| ^a^The World Bank’s governance quality index (<https://info.worldbank.org/governance/wgi> 1996-2018) includes six closely related components: voice and accountability, political stability, government effectiveness, regulatory quality, rule of law, and control of corruption (eigenvalue λ = 5.208, *R^2^* = 0.868; Cronbach’s α = 0.969).  ^b^Perceived livability for oneself, available at [https://www.gallup.com/­analytics/318923/world-poll-public-datasets.aspx](https://www.gallup.com/analytics/318923/world-poll-public-datasets.aspx).  ^c^The logged annual average of individuals per million inhabitants affected by earthquakes, volcanic eruptions, climate extremes, epidemics, insect infestations, and wildfires [retrieved from United Nations Development Programme *Human Development Report 2010* (Palgrave Macmillan, 2010)].  ^d^The prevalence of human-to-human transmitted diseases, based on data from Fincher, C. L., & Thornhill, R. Parasite stress promotes in-group assortative sociality: the cases of strong family ties and heightened religiosity. *Behav. Brain Sci.* **35**, 61-79 (2012).  ^e^The average percentage of employment in the agrarian sector from 1990-2002 [retrieved from Van de Vliert, E. The global ecology of differentiation between us and them. *Nat. Hum. Behav.* **4**, 270-278 (2020)].  ^f^The percentage of the country’s total population living in urban areas [retrieved on 26 August 2018 from <https://www.cia.gov/library/publications/the-world-factbook/fields/349.html>).  ^g^The logged number of inhabitants per square kilometer [retrieved from Parker, P. M. *National cultures of the world: a statistical reference* (Greenwood Press, 1997)].  ^h^The probability that two randomly selected inhabitants of a country belong to different ethnic/religious groups [retrieved from Alesina, A., Devleeschauwer, A., Easterly, W., Kurlat, S., & Wacziarg, R. Fractionalization. *J. Econ. Growth* **8**, 155-194 (2003)].  ^i^Annual net migration rate (negative for emigration, positive for immigration) per 1000 inhabitants during 2010-2015 [retrieved from *World Population Prospects* (<https://population.un.org/wpp/Download/Standard/Migration>) on 10 October 2019]. To reduce extreme kurtosis due to large streams of refugees, the migrant inflow (e.g., Lebanon and Qatar) and migrant outflow (e.g., Syria and Central African Republic) were truncated at 15% of the population.  ^j^Measure of the deviation of the distribution of income among individuals or households within a country from a perfectly equal distribution. [retrieved from United Nations Development Programme *Human Development Report 2010* (Palgrave Macmillan, 2010)]. | | | | | |

**S1 Methods | Analysis scripts for the descriptive geographical model.**

REGRESSION

/MISSING LISTWISE

/STATISTICS COEFF OUTS CI(95) R ANOVA COLLIN TOL CHANGE

/CRITERIA=PIN(.05) POUT(.10)

/NOORIGIN

/DEPENDENT LIVAB

/METHOD=ENTER zLAT

/METHOD=ENTER LATsq

/METHOD=ENTER zLON

/RESIDUALS DURBIN

/CASEWISE PLOT(ZRESID) OUTLIERS(3)

/SAVE COOK.

GRAPH

/SCATTERPLOT(BIVAR)=LATcontr WITH LIVAB

/MISSING=LISTWISE.

GRAPH

/SCATTERPLOT(BIVAR)=LONcontr WITH LIVAB

/MISSING=LISTWISE.

**S2 Methods | Analysis scripts for the explanatory ecological model.**

REGRESSION

/MISSING LISTWISE

/STATISTICS COEFF OUTS CI(95) R ANOVA COLLIN TOL CHANGE

/CRITERIA=PIN(.05) POUT(.10)

/NOORIGIN

/DEPENDENT LIVAB

/METHOD=ENTER zHV

/METHOD=ENTER zNW

/METHOD=ENTER HVxNW

/METHOD=ENTER zLON

/RESIDUALS DURBIN

/CASEWISE PLOT(ZRESID) OUTLIERS(3)

/SAVE COOK.

GRAPH

/SCATTERPLOT(BIVAR)=zHV WITH LIVAB BY NWdichot

/MISSING=LISTWISE.

PROCESS

y= LIVAB

/x=zHV

/m=GQ OHS

/w=zNW

/plot=1

/hc=3

/decimals=F10.3

/center=1

/moments=1

/boot=5000

/conf=95

/model=7.

**S3 Methods | Mplus scripts for the multi-level model.**

VARIABLE:
names =      
 counid DP  WP103 WP105 WP106 WP10250 WP4657 WP1219
 WP1220 WP16 WP3117 WP2319 WP83 WP110
    WP109 WP27 WP65 WP134 WP61 WP128 WP5889 WP5 WAVE
     WP11589 WPID WPID_RANDOM WGT HHWEIGHT2
    YEAR_CALENDAR racial gay immig disab disc ladder
    city_satis education elementary secondary college
    female age housincome_sat eud bus_g helped volun
    help_count learn free respect can_get_ahead
    continen latitude longitud daylength meanT sdT
    rainvari habitatvari wealth socdisc CD CDc PP AS GQ
    PF ET RE IM UR nation_c_sat;


USEOBSERVATION = (WP4657 EQ 1) and (counid /= 162) ;

cluster = counid;
missing = all (-999);


usevariables = disc city_satis secondary college
     female age housincome_sat eud
     habitatvari nation_c_sat wealth GQ   int;

within = city_satis secondary college female age housincome_sat eud ;
between = habitatvari wealth GQ nation_c_sat INT ;

define:
disc = 1-disc;
wealth = LOG (wealth);
age = age/100;

center housincome_sat age eud (groupmean);
center habitatvari wealth nation_c_sat GQ (grandmean);
INT = habitatvari * wealth;


ANALYSIS:

TYPE = TWOLEVEL ;
estimator = bayes;
FBITERATIONS = 5000;
chains = 2;
processors = 4;
BSEED = 18174;
thin = 30;

MODEL:

%within%

disc on female age secondary college housincome_sat eud city_satis;

female age secondary college housincome_sat eud city_satis with
female age secondary college housincome_sat eud city_satis;

%between%
disc;
[disc];


disc on
habitatvari
wealth
INT
GQ (b1)
nation_c_sat (b2);


GQ with nation_c_sat;

habitatvari wealth INT with habitatvari wealth INT;


GQ on habitatvari (a1)
wealth (a2)
INT (a3);

nation_c_sat on habitatvari (c1)
wealth (c2)
INT (c3);

model constraint:
new (ind1 ind2 ind3 ind4 ind5 ind6);
ind1 = a1*b1;
ind2 = a2*b1;
ind3 = a3*b1;
ind4 = c1*b2;
ind5 = c2*b2;
ind6 = c3*b2;


PLOT: TYPE = PLOT2;
OUTPUT: STANDARDIZED tech1 tech8;

**S4 Methods | Measurement of psychosocial flourishing.**

Psychosocial flourishing is an individual-level variable developed on the basis of the annual Gallup World Polls by Mohsen Joshanloo [Optimal human functioning around the world: a new index of eudaimonic well-being in 166 nations. *Br. J. Psychol.* **109**, 637-655 (2018)]. This construct integrates the scientific advantages of parsimony and accuracy. Whereas psychologists overemphasize the heterogeneity problem and the parsimony solution (“never add apples and oranges”), economists overemphasize the homogeneity problem and the accuracy solution (“always add as many fruits as possible”) [for details see: Van de Vliert, E. *Climate, Affluence, and Culture* (Cambridege University Press, 2009, pp. 123-124)]. To do justice to both scientific principles, the composite index of psychosocial flourishing has an optimal trade-off between satisfactory unidimensionality and sufficient heterogeneity, rather than being a homogeneous measure that does not cover the broad nature of the targeted variable.

In Joshanloo’s original study, factor analysis revealed a sufficiently heterogeneous single-factor solution (Cronbach’s *α* = 0.448; eigenvalue λ = 1.631, *R^2^* = 0.233) for the following seven items with a binary response format: Are you satisfied or dissatisfied with your freedom to choose what you do with your life? (factor loading = 0.537); Did you learn or do something interesting yesterday? (0.551); Can people in this country get ahead by working hard, or not? (0.493); [In the past month] have you helped a stranger or someone you didn’t know who needed help? (0.477); [In the past month] have you volunteered your time to an organization? (0.419); If you were in trouble, do you have relatives or friends you can count on to help you whenever you need them, or not? (0.397); Were you treated with respect all day yesterday? (0.484). The construct validity of this individual-level index was confirmed by robust associations with experienced positive affect, satisfaction with the standards of living, being part of the workforce, and being healthy, in this order of importance, and holding true for both men and women.
